# Experimental evidence of vaccine-driven evolution of porcine reproductive and respiratory syndrome virus type 2

**DOI:** 10.1093/ve/veaf056

**Published:** 2025-07-22

**Authors:** Nakarin Pamornchainavakul, Igor A D Paploski, Dennis N Makau, Julia P Baker, Jing Huang, Clarissa P Ferreira, Cesar A Corzo, Albert Rovira, Maxim C-J Cheeran, Samantha Lycett, Andrea Doeschl-Wilson, Declan C Schroeder, Kimberly VanderWaal

**Affiliations:** Department of Veterinary Population Medicine, College of Veterinary Medicine, University of Minnesota, 1365 Gortner Ave, Falcon Heights, MN, 55108, United States; Department of Veterinary Population Medicine, College of Veterinary Medicine, University of Minnesota, 1365 Gortner Ave, Falcon Heights, MN, 55108, United States; Department of Veterinary Population Medicine, College of Veterinary Medicine, University of Minnesota, 1365 Gortner Ave, Falcon Heights, MN, 55108, United States; Department of Public Health, College of Veterinary Medicine, University of Tennessee, 1914 Andy Holt Ave, Knoxville, TN, 37996, United States; Department of Veterinary Population Medicine, College of Veterinary Medicine, University of Minnesota, 1365 Gortner Ave, Falcon Heights, MN, 55108, United States; Department of Veterinary Population Medicine, College of Veterinary Medicine, University of Minnesota, 1365 Gortner Ave, Falcon Heights, MN, 55108, United States; Department of Veterinary Population Medicine, College of Veterinary Medicine, University of Minnesota, 1365 Gortner Ave, Falcon Heights, MN, 55108, United States; Department of Veterinary Population Medicine, College of Veterinary Medicine, University of Minnesota, 1365 Gortner Ave, Falcon Heights, MN, 55108, United States; Department of Veterinary Population Medicine, College of Veterinary Medicine, University of Minnesota, 1365 Gortner Ave, Falcon Heights, MN, 55108, United States; Department of Veterinary Population Medicine, College of Veterinary Medicine, University of Minnesota, 1365 Gortner Ave, Falcon Heights, MN, 55108, United States; Roslin Institute, University of Edinburgh, Easter Bush, Midlothian, EH25 9RG, Edinburgh, Scotland, United Kingdom; Roslin Institute, University of Edinburgh, Easter Bush, Midlothian, EH25 9RG, Edinburgh, Scotland, United Kingdom; Department of Veterinary Population Medicine, College of Veterinary Medicine, University of Minnesota, 1365 Gortner Ave, Falcon Heights, MN, 55108, United States; Department of Veterinary Population Medicine, College of Veterinary Medicine, University of Minnesota, 1365 Gortner Ave, Falcon Heights, MN, 55108, United States

**Keywords:** quasispecies dynamics, within-host evolution, *in vivo* serial passage, longitudinal virome analysis, long-read sequencing

## Abstract

Despite extensive use of vaccination, porcine reproductive and respiratory syndrome virus type 2 (PRRSV-2) continues to evolve, likely driven by escape from natural or vaccine-derived immunity. However, direct evidence of vaccine-induced evolutionary pressure remains limited. Here, we tracked the evolution of PRRSV-2 sublineage 1A strain IA/2014 (variant 1A-unclassified) genome from infection chains of sequentially infected pigs under different immune conditions. Weaned pigs were divided into three groups: a non-immunized control group and two groups vaccinated with different modified live virus (MLV) vaccines, namely Prevacent® PRRS MLV (variant 1D.2) and Ingelvac PRRS® MLV (variant 5A.1). Sixty-four days post-vaccination, the pigs were challenged with IA/2014 PRRSV-2. Virus infection chains (which used serum from pigs in batch *n* to infect batch *n* + 1) were maintained across six sequential batches of roughly seven pigs each, allowing for virus evolution to occur across the ~ 84 days of the infection chain. A total of 110 serum samples were successfully sequenced. Vaccinated groups exhibited over twice the genetic divergence from the original challenge virus (0.3%–0.4% mean nucleotide distance) compared to non-immunized group (0.15%). Variability was concentrated in ORF1a and ORF1b. Deep sequencing revealed more rapid shifts of viral quasispecies composition in vaccinated pigs, and more homogeneous viral populations over batches compared to non-immunized pigs. Selection pressure analyses indicated strong purifying selection in one vaccinated group, though without clear signals at known antigenic sites in all treatment groups. However, vaccinated pigs had significantly higher cycle threshold values (*P*<.001), indicating lower viral loads and suggesting potential fitness limitations for highly diverged viruses in immunized pigs. These findings demonstrate that MLV vaccination can exert substantial evolutionary pressure on PRRSV-2, driving genetic diversification and highlighting the need for continuous PRRS monitoring and adaptive control strategies.

## Introduction

Elimination of porcine reproductive and respiratory syndrome (PRRS), a costly disease undermining global pork production (Holtkamp et al. 2013, Nathues et al. 2017), remains an elusive goal because the effectiveness of vaccination—the primary control measure—is continually complicated by the emergence of new genetic variants of the rapidly mutating PRRS virus (PRRSV) (Lunney et al. 2016, Nan et al. 2017, Paploski et al. 2021). In the USA, where PRRS cases are mainly caused by PRRSV-type 2 (PRRSV-2), more than half of breeding herds experience annual outbreaks or ongoing circulation of the virus (Morrison Swine Health Monitoring Project 2024). Thus, a high percentage of commercial swine have some level of immunity to PRRSV-2, whether through natural infection, vaccination, or field virus inoculation. Despite high levels of population immunity, PRRSV-2 persists and proliferates. Each year, ~4 new PRRSV-2 genetic variants (i.e. groups of viruses whose ORF5 gene are ~ 5% different from other field viruses on average) are identified, some of which are highly virulent and can increase in prevalence by over 200% in a single year (Kikuti et al. 2021, VanderWaal et al. 2024). These field data contribute to ongoing discussions about a potential link between vaccine use and PRRSV-2 evolution, especially considering that PRRSV immunity is not fully protective and secondary exposures are common in the field.

PRRSV-2 is a positive-sense, single-stranded RNA virus belonging to the family *Arteriviridae*, within the order *Nidovirales* (Anon n.d.). Its genome comprises at least 10 open reading frames (ORFs), with ORF1a and ORF1b encoding polyproteins that are subsequently cleaved into 14 non-structural proteins (nsps) essential for viral replication and transcription. The remaining ORFs encode structural proteins, including minor envelope proteins (GP2, E, GP3, GP4 from ORF2a, ORF2b, ORF3, and ORF4, respectively), major envelope proteins (GP5 and M from ORF5 and ORF6, respectively), and the nucleocapsid protein (N) from ORF7 (Wissink et al. 2005, Snijder et al. 2013, Lunney et al. 2016). The viral surface envelope proteins play a direct role in host cell attachment and entry (Das et al. 2010, Van Breedam et al. 2010, Kappes and Faaberg 2015). Based on analysis of ORFs 3, 4, and 5, PRRSV-2 has been estimated to have exceptionally high evolutionary rates (4.7–9.8 × 10^−2^ substitutions/nucleotide site/year, or s/n/y), significantly higher than the typical range for RNA viruses (10^−3^–10^−5^ s/n/y) (Hanada et al. 2005), though evolutionary rate and genetic variation of PRRSV-2 differ across genomic regions and subtypes (Tian et al. 2007, Veit et al. 2014, Guo et al. 2021, Paploski et al. 2021, Pamornchainavakul et al. 2022). Multiple selection pressure analyses on field datasets consistently suggest that PRRSV-2 evolves under diversifying selection, particularly at immunogenic sites, reflecting its adaptability in evading host immunity (Hu et al. 2009, Delisle et al. 2012, Chen et al. 2016, Do et al. 2016, Paploski et al. 2019, Rupasinghe et al. 2022).

Currently, modified live virus (MLV) vaccines are commonly used to immunize pigs against PRRSV-2, as they provide clinical protection and are commercially available (Zhang et al. 2023). However, there are concerns about potential for recombination with field strains (Li et al. 2009, Bian et al. 2017, Wang et al. 2019) and reversion to virulence (Nielsen et al. 2001, Kimman et al. 2009). MLVs also offer only limited cross-neutralizing protection, as evidenced by the varying levels of partial immunity they provide against genetically diverse strains (Lager et al. 1999, Kimman et al. 2009, Huang and Meng 2010, Vu et al. 2017). This limitation presents a significant challenge, potentially allowing PRRSV-2 to persist and continue to evolve. Furthermore, PRRSV-2 is known to employ immune evasion mechanisms, such as glycan shielding through N-glycosylation (Ansari et al. 2006, Vu et al. 2011), and the presence of immunodominant non-neutralizing (decoy) (Lopez and Osorio 2004, Thaa et al. 2013, Huang et al. 2025) or strain-specific neutralizing epitopes (Popescu et al. 2017) on the GP5 protein. Given these factors, the imperfect protection offered by MLVs not only hinders PRRS control efforts by failing to keep pace with the virus’s rapid adaptability but also raises concerns that partial immunity conferred by vaccine may exert evolutionary pressure on PRRSV-2.

Selection pressures induced by vaccination have been observed in several viral diseases with imperfect vaccines. Marek’s disease virus in chickens is a notable example, where the selection of highly virulent strains was observed in vaccinated populations that survived (Read et al. 2015). Like PRRSV, vaccination reduces clinical symptoms of Marek’s disease but allows the virus to continue spreading among the population (Read et al. 2015, Bull and Antia 2022). For human seasonal influenza, the ladder-like phylogenetic trees of the hemagglutinin and neuraminidase proteins reflect continuous positive selection on antigenic evolution driven by host immunity, whether conferred by natural infection or, in some cases, experimentally shown to be driven directly by vaccine pressure (Wolf et al. 2006, Hoelzer et al. 2010, Debbink et al. 2017). This phenomenon has not been definitively demonstrated in diseases like SARS-CoV-2, but it remains a major concern due to the virus’s rapid evolution and global scale of vaccination efforts (Rouzine and Rozhnova 2023). Similar to these viruses, PRRSV-2 possesses several key traits, such as a high evolutionary rate, high transmissibility, and incomplete vaccine-induced protection, which could support evolution under vaccine-driven immune pressure, although conclusive evidence is still lacking. In this study, we experimentally assess whether MLV vaccination can drive PRRSV-2 evolution by comparing the evolutionary dynamics of the virus in an *in vivo* infection chain between non-immunized and vaccinated groups under controlled conditions. We hypothesized that partial immunity from the MLV vaccines exerts selection pressure on the virus, leading to more rapid genetic divergence. We analysed genetic changes at both the consensus genome and quasispecies levels. The novel insights from our findings are crucial for both the future development of vaccines and the improvement of current vaccination strategies.

## Methods

### Ethic statement

The animal and biosafety work for this study, including the collection and handling of samples, was approved by the University of Minnesota Institutional Animal Care and Use Committee (IACUC) under Protocol ID: 2012-38696A, and the Institutional Biosafety Committee under Protocol ID: 2201-39740H. The study was conducted in accordance with federal/state legislation, institutional requirements, and the Guide for the Care and Use of Laboratory Animals of the National Institutes of Health.

### Sourcing of animals and vaccination protocol

**Figure 1:**
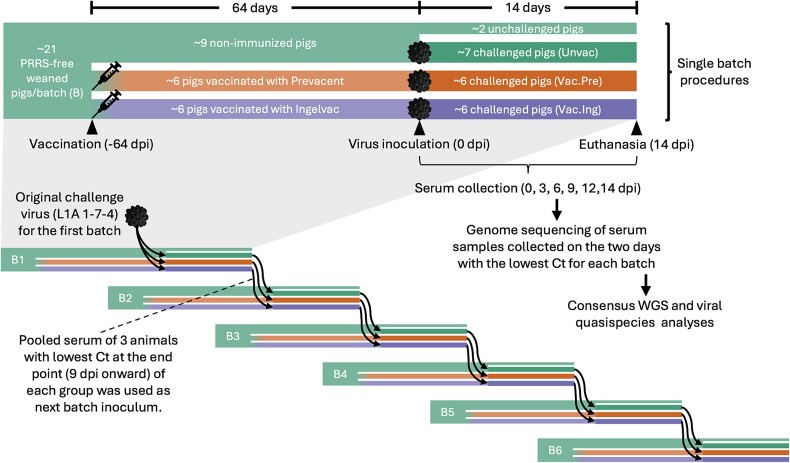
Experimental design of the infection chain experiment. (Top) The timeline of detailed procedures for each batch of ~ 21 animals. (Bottom) Diagram depicting all six batches of the infection chain spanning ~84 days, showing the original challenge virus (IA/2014 PRRSV-2 L1A 1-7-4) in the first batch and the pooled serum used to infect subsequent batches within the same treatment group. Each batch began with weaned pigs of the same age, although the starting time points differed.

A total of 127 female pigs (~21 pigs per batch) were included in this study. The animals were sourced from a PRRS-negative herd that met the criteria for being pathogen-free (free from PRRSV, swine influenza virus, *Mycoplasma hyopneumoniae*, and other respiratory pathogens). At 21–28 days of age (post-weaning), the pigs were initially housed at the research facilities of Swine Services Unlimited, Inc. Weaned pigs were randomly assigned to three groups that were housed in separate Animal Biosafety Level 2 (ABSL-2) rooms. Group 1 contained non-immunized animals (*n* = 7–10 pigs per batch), while Groups 2 and 3 (*n* = 6–7 pigs per batch) included animals vaccinated with either Prevacent® PRRS MLV (Elanco US, Inc.), a sublineage 1D (variant 1D.2) strain, or Ingelvac PRRS® MLV (Boehringer Ingelheim Animal Health USA Inc.), a sublineage 5A (variant 5A.1) strain based on ORF5 classification (Yim-im et al. 2023, VanderWaal et al. 2024). Vaccines were administered immediately after segregated housing following the dosage and administration instructions provided by the product’s manufacturer instructions. Sixty days post-vaccination (at ~3 months of age), the animals were transferred to the veterinary isolation facility (VIF) at the University of Minnesota (Fig. 1). They were acclimated for 3 days at the VIF before being challenged with a PRRSV-2 virus (see below). Before the challenge, serum samples from all animals were tested by the University of Minnesota Veterinary Diagnostic Laboratory (VDL) using reverse transcription polymerase chain reaction (RT-PCR) with VetMAX PRRSV 3.0 Reagents (Thermo Fisher Scientific, USA) to confirm the absence of PRRSV-2 or MLV, with a cycle threshold (Ct) value >35 indicating a negative result. The challenge at 64 days post-vaccination was timed to ensure that animals were no longer viremic with the MLV strain (as confirmed by negative RT-PCR results), and to elicit an anamnestic (memory) response once neutralizing antibody levels had waned, which was confirmed by higher neutralizing antibody levels in Batch 1 of vaccinated animals to both lineage 5 and sublineage 1A viruses at Day 14 post-inoculation (14 dpi) compared to prechallenge (0 dpi) (Huang et al. 2025).

### Challenge virus used for initial inoculum

A sublineage 1A RFLP 1-7-4 PRRSV strain IA/2014 (variant 1A-unclassified, GenBank accession no. MZ423533.1) (Schroeder et al. 2021) was propagated in MARC-145 cells (RRID: CVCL_4540) cultured in minimum essential medium (MEM; Gibco™, Waltham, MA) supplemented with 10% foetal bovine serum (Gibco) and 10 mM HEPES (Gibco). When > 90% cytopathic effect was observed, the infected cell culture underwent a single freeze–thaw cycle, and the supernatant was collected following centrifugation at 400 × *g* for 5 min. For viral titration, 0.1 ml of the stock virus was serially diluted 10-fold from 10^−1^ to 10^−10^, and 0.1 ml of each dilution was inoculated into MARC-145 cells in 96-well plates, with eight replicate wells per dilution. Infection was confirmed by immunostaining with a PRRSV nucleocapsid-specific antibody (SR30-A, RTI, Brookings, MD). Viral titers, expressed as the 50% tissue culture infectious dose (TCID_50_/ml), were determined using the Reed–Muench method (Reed and Muench 1938). The propagated virus was then subjected to whole-genome sequencing to confirm its genetic consistency with the original strain.

### Viral challenge design

*In vivo* PRRSV-2 infection chains were established for the three experimental groups across six consecutive batches of pigs (Fig. 1). An additional group of two randomly selected animals from the non-immunized group served as unchallenged controls for each batch. At the VIF, the three experimental groups plus the unchallenged controls were housed in four separate ABSL-2 isolation units. Except for the unchallenged controls, all pigs were inoculated intramuscularly with 10^5^ TCID_50_ of PRRSV-2 sublineage 1A, isolate IA/2014 (Schroeder et al. 2021) (SRA accession no. SRR32380994) per animal on Day 0 post-inoculation (0 dpi). Blood samples (10 ml) were collected from the jugular vein of each pig using vacutainers at 0, 3, 6, 9, and 12 dpi. On Day 14 dpi, 100 ml of blood was collected from each animal, after which the animals were euthanized (see ‘single batch procedures’ in Fig. 1). Blood samples were stored in ice-filled containers and centrifuged within 3 h of collection at 5000 × *g* for 15 min. Serum was then aliquoted into Eppendorf vials for further use.

For all batches, serum samples from all animals were submitted for PRRSV-2 RT-PCR testing at the University of Minnesota VDL. From 9 dpi onward, the three animals with the lowest Ct values on a single day, typically the day of euthanasia (14 dpi), were selected to generate the inoculum for the next batch. In cases where an entire group of animals required early euthanasia due to severe clinical symptoms, samples collected on that day (at any dpi) were used as the inoculum for the subsequent batch. Ultimately, all inocula came from 14 dpi serum samples, except for Batch 3 (9 dpi) of all treatment groups and the challenged non-immunized group in Batches 4 and 5 (10 dpi). The next batch of animals was transferred to the VIF within 3 days of the euthanasia day of the previous batch. For all batches, procedures and timelines were the same as in the first batch (Fig. 1).

The lowest Ct value observed in each individual animal was also used as an indirect measure of viral load and was used to evaluate differences in viral load across groups and batches, employing the Kruskal–Wallis test and Dunn’s post-hoc test from the *FSA* v0.9.6 R package (Ogle et al. 2025).

### RNA extraction, cDNA synthesis, and ONT sequencing

Viral RNA was extracted from 200 μl of serum collected from all animals on the 2 days with the lowest Ct values for each batch utilizing the NucleoMag® Virus kit (TaKaRa Bio USA, Inc.) in conjunction with the KingFisher Flex Magnetic Particle Processor (Thermo Fisher Scientific, USA), following the manufacturer’s recommended protocol. For cDNA synthesis, first-strand generation was carried out using the Template Switching RT Enzyme Mix (New England Biolabs), adhering to the procedure outlined by Schroeder et al. (2021), with the modification that a pool of primers specific to PRRSV-2 lineage 1 genomes was employed (Supplementary Table 1). The reaction setup included 4 μl of extracted RNA, 1 μl of a 10 μM pooled primer mix, and 1 μl of 10 mM dNTPs. The thermal profile consisted of a 5-min incubation at 70°C, rapid cooling to 4°C, followed by the addition of 2.5 μl of Template Switching Buffer, 0.5 μl of 75 μM template switching oligonucleotide (TSO) (sequence: AAGCAGTGGTATCAACGCAGAGTACrGrGrG), and 1 μl of Template Switching Enzyme. First-strand synthesis was performed at 42°C for 90 min, then 85°C for 5 min, with the final hold at 4°C until subsequent processing.

Second-strand cDNA synthesis was performed in a 25 μl reaction containing 2 μl of first-strand product, 0.25 μl of PrimeSTAR HS Polymerase (2.5 U/μl), 5 μl of 5× PrimeSTAR buffer, 2 μl of 2.5 mM dNTPs (TaKaRa Bio USA, Inc.), 1 μl of 10 μM TSO primer (sequence: AAGCAGTGGTATCAACGCAGAGTAC), and 14.75 μl of molecular-grade water. PCR cycling conditions were 94°C for 1 min, followed by 30 cycles of 98°C for 10 s, 60°C for 15 s, and 68°C for 2 min 30 s, with a final hold at 4°C.

Purification of cDNA was achieved using CleanNGS DNA & RNA Clean-Up Magnetic Beads, A 1:1 bead-to-sample ratio was maintained by mixing 25 μl of beads with 25 μl of PCR-amplified cDNA. The mixture was incubated at room temperature for 10 min before magnet-assisted separation. The beads were washed twice with 200 μl of 80% ethanol, air-dried for 1 min, and resuspended in 12 μl of molecular-grade water for 10 min. Following magnetic separation, the eluted cDNA was quantified using the Qubit 1× dsDNA HS Assay on a Qubit® 4.0 Fluorometer according to the manufacturer’s protocol.

The cleaned cDNA products were used to generate sequencing libraries with the Rapid Barcoding Sequencing Kit (SQK-RBK114.24), following the manufacturer’s guidelines (Oxford Nanopore Technologies, UK). Libraries were prepared separately for each experimental group to prevent cross-contamination and loaded onto separate flow cells. A different flow cell was used for each of the following groups: non-immunized challenged control group and the two vaccinated groups. Sequencing was performed on R.10 flow cells (FLO-MIN114) using a GridION platform (Oxford Nanopore Technologies, UK) with default settings for 24 h.

### Genome assembly and consensus genome analysis

The raw reads from all sequenced samples and the original challenge virus (BioProject accession no. PRJNA1224032) were preprocessed using adapter trimming (default settings) with Porechop v0.2.4 (Wick et al. 2017) and quality-based filtering (read average quality score > 10) with NanoFilt v2.8.0 (De Coster et al. 2018). The processed reads were then mapped to the reference sequence (GenBank accession no. MZ423533.1) (Schroeder et al. 2021) using minimap2 v2.26 (Li 2021). Subsequently, SAMtools v1.21 (Danecek et al. 2021) was employed to convert the mapped read alignment into a binary alignment map (BAM) file, which served as input for consensus whole-genome sequence (WGS) generation. This process was performed using an in-house Python (Van Rossum et al. 2009) script (https://github.com/NakarinP/PRRSV-2-infection-chain-supplementary-materials/blob/master/bamtoconsensus.py), ensuring that the consensus nucleotide at each position was derived from a minimum depth of five reads (Ladner et al. 2014) and not from the reference sequence. The consensus genomes of all samples were aligned using the local pairwise alignment approach (L-INS-i) in MAFFT v7.526 (Katoh 2002). Samples with consensus genomes containing gaps or missing nucleotides exceeding 20% of the full-length genome alignment (Wiens 2006) were excluded from further analyses.

The genetic relationships between viruses from each treatment group and the original challenge virus were examined through phylogenetic analysis and pairwise genetic distance comparisons. Three maximum likelihood phylogenetic trees were reconstructed, one for each treatment group and the challenge virus, using the best-fit substitution models: HKY + F + I + G4 for the non-immunized group, K3Pu + F + I + G4 for the Prevacent group, and TPM2 + F + I for the Ingelvac group. The models were automatically selected by ModelFinder (Kalyaanamoorthy et al. 2017) based on the Bayesian information criterion, and the trees were constructed with 1 000 ultrafast bootstrap replicates using IQ-TREE v2.3.0 (Minh et al. 2020). Trees were re-rooted on the original challenge virus and visualized with ggtree v3.8.2 (Xu et al. 2022). This approach avoided misinterpreting direct ancestral links between samples from different groups, which a unified tree of samples from all groups might imply. Pairwise genetic distances between all viral genomes were calculated using the ‘dist.dna’ function (K80 model) and transformed into coordinates with the ‘pcoa’ function in ape v5.8 (Paradis and Schliep 2019). The principal coordinate analysis (PCoA) plot and the mean genetic distances—both between each batch of each group and the challenge virus, and within each batch of each group—as well as results from subsequent analyses were visualized using the *ggplot2* R package v3.5.1 (Ginestet 2011). Dispersion from the challenge virus was quantified by calculating the range of Euclidean distances (maximum − minimum distance) in PCoA’s PC1–PC2 coordinate space for each treatment group. Statistical significance of distinct clustering was assessed using permutational multivariate analysis of variance (PERMANOVA) implemented in the *vegan* R package v2.6.8 (Oksanen 2010), to test for differences in group centroids. The association between genetic distance from individual samples to the challenge virus and various experimental and sequencing quality related factors was assessed using a generalized linear model (GLM) with a gamma distribution and log link, as the data closely followed the theoretical gamma distribution based on the Cullen and Frey diagnostic plot (Cullen and Frey 1999, Delignette-Muller and Dutang 2015). The model included treatment group, batch, their interaction (treatment × batch), collection time point (dpi), Ct value, percentage of genome coverage, and number of mapped reads as predictors. The analysis was conducted using the R stats package v4.3.1 (R Core Team 2018). To identify genomic regions contributing to overall diversity, we calculated normalized Shannon entropy (Shannon 1948) (0 = fully conserved, 1 = highly diverse) to measure site-wise genetic variability from the genome alignment of each group.

### Viral quasispecies diversity analysis

Read alignments of each viral sample from the genome assembly step was segmented into genes or protein-coding regions based on the reference genome annotation. The inclusion criteria for individual samples to enter subsequent viral quasispecies analyses (alpha and beta diversity, and selection pressure analysis) included having a depth of at least 100 continuous long reads covering that genomic region (Supplementary Table 2). Additionally, genomic regions included in subsequent analyses must exceed 300 nucleotides in length and have at least 30% of the total samples (representing at least 15 of the 18 batches across all treatments groups) that meet the minimum depth for inclusion. These criteria ensured that selected samples and regions sufficiently represented both the within-host viral population diversity and the infected animals across the span of the infection chain.

Quantification of viral quasispecies diversity within each sample was performed using the QoALa workflow implemented in the *longreadvqs* R package v0.1.3 (Pamornchainavakul et al. 2024). Given the high sequencing error rates inherent to nanopore technology, single nucleotide variants (SNVs) with a frequency below 5% of the total read depth were replaced with the majority nucleotide at those positions, as suggested by Pamornchainavakul et al. (2024). This threshold was determined through an optimization process (see Supplementary Fig. 1), in which low-frequency SNVs—originally contributing to 100% singleton haplotypes in most samples—were sequentially replaced at increasing frequency cutoffs (from 0% upward). The chosen 5% threshold corresponded to the ‘elbow point’, where the percentage of singleton haplotypes plateaued, indicating a stable reduction in artifactual diversity likely caused by sequencing errors. After minimizing sequencing noise, read alignments were down-sampled to a uniform depth of 100 reads per sample for each genomic region to ensure diversity analyses was based on the same number of reads across samples; down-sampling to this depth has been shown to not alter values for the diversity metrics utilized in this study (Pamornchainavakul et al. 2024). Viral subpopulations within each sample were analysed on two scales: haplotypes, defined as groups of reads with identical nucleotide sequences; and operational taxonomic units (OTUs), which represent groups of haplotypes sharing closely related genetic profiles. OTUs were defined through the QoALa workflow based on the SNV distance matrix of haplotypes using multidimensional scaling and *k*-means clustering, classifying haplotypes into 10 OTUs per each genomic region.

Quasispecies composition was investigated and compared for each genomic region in terms of alpha and beta diversity (i.e. within and between samples, respectively). For alpha diversity, the Gini-Simpson index was used to quantify diversity and evenness of haplotypes within each sample (Gregori et al. 2016). This index ranges from 0 (no diversity, where only a single haplotype exists) to 1 (maximum diversity with infinitely many equally abundant haplotypes). This metric is not sensitive to down-sampling of reads (Pamornchainavakul et al. 2024). Given that the Gini-Simpson index follows a beta distribution, we fit a beta regression model to the data using the *betareg* R package v3.2.1 (Cribari-Neto and Zeileis 2010). In the model, the Gini-Simpson index was the response variable, and predictors included treatment group, batch, their interaction (treatment × batch), collection time point (dpi), Ct value, full sequencing depth (before down-sampling to 100 reads).

For beta diversity, the dissimilarity between two sample’s OTU compositions (i.e. differences in the make-up and proportional abundances of groups of similar haplotypes) was measured for each genomic region using the Bray–Curtis dissimilarity index (Bray and Curtis 1957), which ranges from 0 to 1. In this analysis, a value of 0 indicates that two samples shared identical proportions of each OTU, whereas a value of 1 signifies that the two samples had no OTUs in common. OTUs were used for beta diversity analysis, because direct comparison of numerous highly similar haplotypes across samples would overestimate diversity. Grouping these into OTUs offers a more accurate and biologically meaningful representation of genetic relatedness between samples. The dissimilarity matrix for all samples and the original challenge virus was calculated using the ‘vegdist’ function from the *vegan* R package v2.6.8 (Oksanen 2010). The mean dissimilarity of OTU compositions was summarized for samples in each batch of each group relative to the original challenge virus, as well as within group of samples from each batch of each group.

Given the distribution of the Bray–Curtis dissimilarity index, beta regression (Cribari-Neto and Zeileis 2010) was used to model the index between individual samples and the challenge virus, with the same set of predictors as the Gini-Simpson analysis: treatment group, batch, the treatment × batch interaction, dpi, Ct value, and full sequencing depth. To evaluate differences in diversity across groups and batches, we used PERMANOVA, fitting a model on the dissimilarity matrix with treatment group, batch, and their interaction as independent variables. This analysis was performed using the ‘adonis2’ function in the *vegan* package (Oksanen 2010). *Post-hoc* multilevel pairwise comparisons between groups and batches were conducted using the *pairwiseAdonis* R package v0.4.1 (Martinez 2020).

### Selection pressure analysis

Amino acid mutations over the course of the experiment for each treatment group were quantified through selection pressure analysis. From the aligned reads used in the viral quasispecies analysis, 100 unique haplotypes (or all haplotypes if fewer than 100 were available) were randomly selected per gene, per batch, and per treatment group, as well as for the original challenge virus. Haplotypes containing stop codons within coding regions were excluded prior to analysis. Haplotype gene sequence alignments for each group, along with the challenge virus, were analysed using three site-wise selection pressure methods available on the Datamonkey web application (Weaver et al. 2018): fixed effects likelihood (FEL) (Kosakovsky Pond and Frost 2005), Fast Unconstrained Bayesian AppRoximation (FUBAR) (Murrell et al. 2013), and mixed effects model of evolution (MEME) (Murrell et al. 2012). All methods estimate synonymous (dS) and non-synonymous (dN) substitution rates per site to test whether a site evolved under purifying (dN < dS), neutral (dN = dS), or diversifying selection (dN > dS). FEL uses a maximum likelihood approach to test if dN significantly differs from dS (Kosakovsky Pond and Frost 2005), FUBAR employs a Bayesian framework with posterior probabilities to detect diversifying selection (Murrell et al. 2013), and MEME identifies episodic diversifying selection by modelling variations in dN and dS across branches and sites (Murrell et al. 2012). Codon sites found to be under significant selection pressure (*P*-value < .05 for FEL and MEME, or posterior probability > 0.9 for FUBAR) in at least two of the three tests were summarized and compared across treatment groups.

## Results

### Relative viral load in serum was highest in non-immunized animals

Within 3 days before challenge, all animals from all treatment groups tested negative for PRRSV-2, confirming the absence of background infection. Following the challenge, the estimated virus quantity in the non-immunized challenged control group (Unvac) was significantly higher than in both the group vaccinated with Prevacent MLV (Vac.Pre) and the group vaccinated with Ingelvac MLV (Vac.Ing) across all batches. This was determined by comparing the lowest Ct value for each animal during the challenge periods (Supplementary Figs 2 and 3), and a Kruskal–Wallis test revealed significant differences in Ct values across treatments (*P* < .001) and batches (*P* < .001). Dunn’s post-hoc test confirmed significant Ct differences between Unvac and Vac.Pre (*P* < .001) and Unvac and Vac.Ing (*P* < .001), while no difference was observed between the two vaccinated groups (*P* = 1). For batches, significant differences were identified between Batch 1 and Batches 4 through 6 (*P* < .001), as well as between Batch 3 and Batches 4 through 6 (*P* < .05).

### Higher levels of evolutionary change were observed in the viral genomes from vaccinated groups

A total of 110 consensus WGSs were successfully assembled and passed the quality control step. These included the original challenge virus (ChV; *n* = 1 genome), the Unvac group (*n* = 44), the Vac.Pre group (*n* = 39), and the Vac.Ing group (*n* = 26). The median number of WGSs per batch was 8 (interquartile range [IQR]: 5–10.25) for the Unvac group, 6 (IQR: 4–7.75) for the Vac.Pre group, and 5 (IQR: 3—6.5) for the Vac.Ing group. All consensus WGSs from the Unvac group’s first batch contained > 20% missing nucleotides or gaps and were excluded from analysis (Supplementary Table 2). While the exact cause of their lower sequencing quality is unclear, consistent sequencing protocols across samples suggest it likely resulted from mishandling or suboptimal storage leading to RNA degradation.

**Figure 2:**
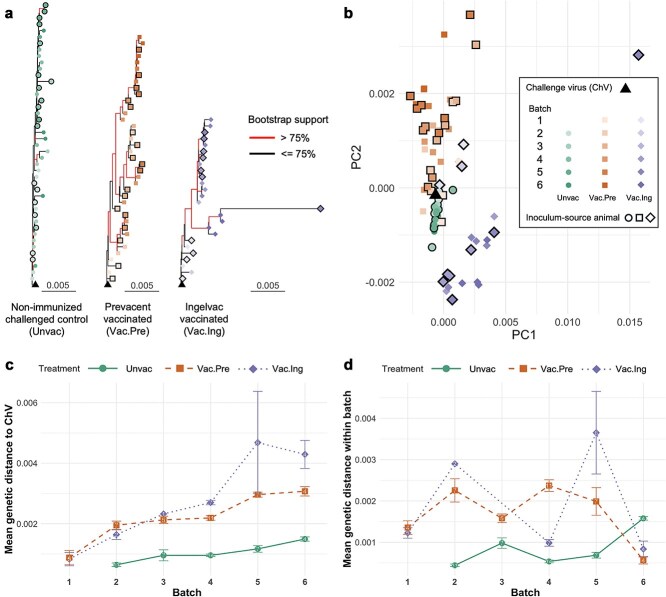
IA2014 PRRSV-2 genomic variation across treatment groups based on consensus genomes. (a) Maximum likelihood phylogenetic trees of the three treatment groups, rooted on the challenge virus. Branches with bootstrap clade support > 75% are highlighted. (b) PCoA plot of pairwise distances between all sequenced samples. The shapes of tip points in (a) and scatter points in (b) indicate the treatment groups: triangle for the challenge virus, circle for the non-immunized challenged control, square for the Prevacent-vaccinated group, and diamond shape for the Ingelvac-vaccinated group. Additionally, shade intensity reflects the batch in the infection chain (with darker shades indicating later batches), and a border around the shapes indicates that the sequence was derived from an animal whose serum was used to produce the inoculum for the next batch. (c) Mean genetic distance of each batch relative to the challenge virus. (d) Mean genetic distance within each batch of each treatment group. Error bars in (c) and (d) represent standard error, while numbers on data points indicate sample sizes (detailed sample sizes are provided in Supplementary Table 2).

The overall dynamics of PRRSV-2 IA/2014 genetic diversity between non-immunized and vaccinated animals differed noticeably, as evidenced by consistent results from various genetic comparative approaches. According to the maximum likelihood phylogenetic trees, most samples from the earliest available batch of all groups were positioned at the base of the tree, close to the challenge virus. However, the unvaccinated group’s tree exhibited multiple polytomies and relatively short branch lengths with bootstrap clade support generally lower than 75%, all of which suggests minimal divergence from the original challenge virus. In contrast, both the Vac.Pre and Vac.Ing groups showed longer branch lengths and demonstrated at least two distinct ladder-like clades in the later batches, with bootstrap support over 75% (Fig. 2a).

When comparing all treatment groups using PCoA based on a pairwise genetic distance matrix, samples from the unvaccinated group tightly clustered near the challenge virus. In contrast, samples from both vaccinated groups were more widely dispersed, spanning 3.48-fold (Vac.Pre) and 12.39-fold (Vac.Ing) greater ranges in distance from the challenge virus compared to the unvaccinated group along the first two principal coordinates (PC1 and PC2), extending in different directions and forming distinct clusters (PERMANOVA: *P* < .0001 for all comparisons; Supplementary Table 3), with one outlier from Batch 5 of the Vac.Ing group (Fig. 2b).

Considering the changes in genetic diversity across the sequential batches in the infection chain, viruses from the vaccinated groups had a low mean genetic distance to the challenge virus in the earliest batch, ranging from 0.083% to 0.088%. Across batches, the mean distance in the vaccinated groups increased to 0.31%–0.43% by the final batch. In contrast, the mean distance for viruses from the unvaccinated group remained lower throughout, starting at 0.064% in the second batch and reaching only 0.15% by the endpoint (Fig. 2c). The GLM analysis also revealed that batch (coefficient = 0.197, *P* < .001) and treatment group were significantly associated with genetic distance from individual samples to the challenge virus. The Vac.Pre group showed a significant positive association (coefficient = 0.833, *P* = .004), with a significant batch × Vac.Ing interaction (coefficient = 0.145, *P* = .041). Other factors (dpi, Ct value, genome coverage, mapped reads) were not significant (all *P* > .05) (Supplementary Table 4). Within-batch diversity fluctuated across batches for all treatment groups, with no consistent differences across treatments or over batches (Fig. 2d).

Site-wise variability of consensus genomes revealed that variable sites were predominantly concentrated in ORF1a and ORF1b. Notably, viruses from vaccinated groups exhibited hotspots of polymorphism (continuous sites with normalized Shannon entropy > 0.25), including the nsp1–nsp2 junction, distal nsp2, nsp7, and distal nsp9, none of which were observed in the unvaccinated group. However, many of these highly variable regions coincided with areas of low sequencing depth (<100 nucleotides/site) in vaccinated groups (Supplementary Fig. 4).

### Viral quasispecies diversity within samples (alpha diversity) showed no consistent patterns related to vaccination or batch

**Figure 3:**
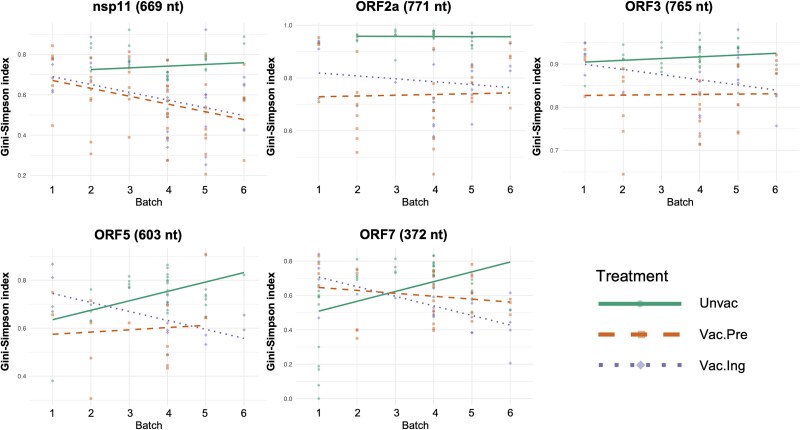
Gini-Simpson index of within-sample haplotype diversity across genomic regions. Scatter points represent the Gini-Simpson index for each sample across different batches, with a regression line indicating trends over batches. Colour and shape of points and lines are coded by treatment group. The nucleotide length of each genomic region is shown in parentheses.

Deep sequencing data from five genomic regions—nsp11 in ORF1b, ORF2a, ORF3, ORF5, and ORF7—had sufficient data for viral quasispecies analysis (see Methods for selection criteria). We measured haplotype diversity within each sample using the Gini-Simpson index. Overall, regression analysis of the effect of treatment and batch on haplotype diversity was variable, and there was no consistent pattern across genes (Table 1 and Fig. 3).

**Table 1:** Coefficients of variables in the beta regression models for the Gini-Simpson index of within-sample haplotype diversity across observed genomic regions. Separate models were fit for each gene/region.

Gene/region	Vac.Ing	Vac.Pre	Batch	Vac.Ing × Batch	Vac.Pre × Batch	dpi	Ct	Full read depth
nsp11	0.0887	−0.4456	0.0693	−0.277^*^	−0.1535	−0.0228	0.0292	−0.0001
ORF2a	−1.2777	−1.6078^*^	0.0184	−0.0949	−0.0397	−0.0351	0.0153	−0.0003^*^
ORF3	−0.0736	−0.6994	0.0829	−0.1573	−0.0878	−0.0235	0.0337	−0.0001
ORF5	−0.0797	−0.7457	0.1069	−0.1921	−0.0176	−0.0218	−0.0156	−0.0006^**^
ORF7	0.173	−0.4874	−0.0488	−0.2261^*^	−0.0131	0.0312	0.05^*^	0.0003^*^

Significance of coefficients: ^*^P < .05; ^**^P < .01; ^***^P < .001.

For ORF2a, the Vac.Pre group exhibited significantly lower haplotype diversity compared to the non-immunized group (***P* < .05**). For nsp11 and ORF7, haplotype diversity in the Vac.Ing group significantly decreased in the later batches, as shown by significant negative interaction terms between Vac.Ing and batch (***P* < .05**). No significant vaccination-related effects were observed for ORF3 or ORF5. Though full sequencing depth was significant for ORF2a (***P* < .05**), ORF5 (***P* < .01**), and ORF7 (***P* < .05**), and Ct value was significant for ORF7 (***P* < .05**), the magnitude of these technical effects was much smaller than the treatment and batch effects. These variable findings lead us to conclude that there were no clear patterns related to vaccination on within-sample haplotype diversity (Table 1).

### Viral quasispecies composition was increasingly dissimilar from the challenge virus in the vaccinated groups compared to the non-immunized group

Haplotypes within each genomic region were grouped by genetic relatedness into 10 OTUs, and OTU composition for each sample was calculated as the proportional occurrence of each OTU. Across five genomic regions, defining SNV positions between OTUs (the smallest number of positions that can distinguish all OTUs) range from 10 to 150 and consensus sequence differences between OTUs range from 3.31 to 34.13 SNVs (ORF3 and ORF5 showing the highest values for both), while within-OTU diversity remains consistent at 4.39–5.23 SNVs between haplotypes, demonstrating variable discriminatory power between regions while maintaining consistent internal cohesion within OTUs (see Supplementary Table 5 for a summary and detailed list of OTU defining SNVs for each genomic region, and Supplementary Figs 7–16 for visualizations of haplotype distributions and OTU proportions across treatment groups and batches for each genomic region).

**Figure 4:**
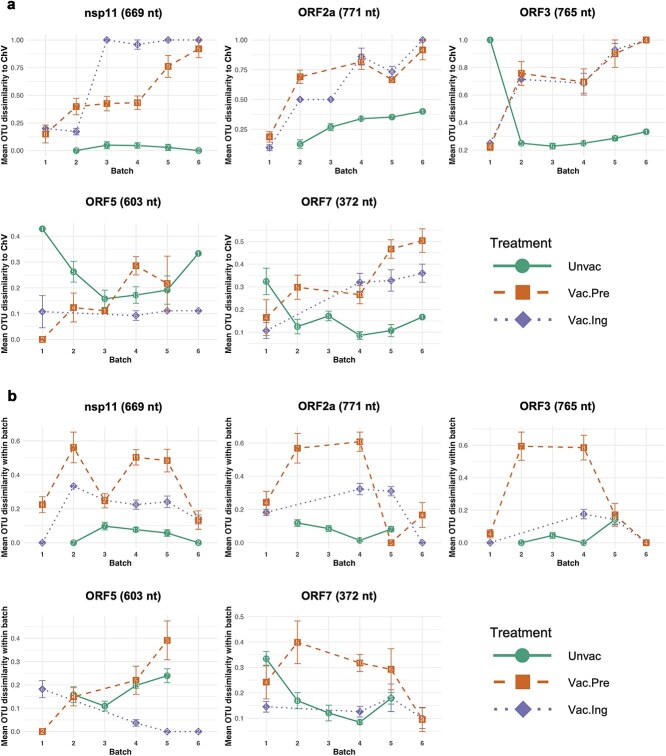
Mean Bray–Curtis dissimilarity of OTU composition across genomic regions. (a) Mean OTU dissimilarity relative to the original challenge virus. (b) Mean OTU dissimilarity within each batch of each treatment group. Error bars in (a) and (b) represent standard error, while numbers on data points indicate sample sizes (detailed sample sizes are provided in Supplementary Table 2). Colour and shape of points and lines are coded by treatment group. The nucleotide length of each genomic region is shown in parentheses.

Bray–Curtis dissimilarity was used to compare the similarity of OTU compositions between samples. Vaccinated groups showed steadily increasing dissimilarity from the original challenge virus across batches for most genes (except ORF5), with some regions reaching 100% dissimilarity by the endpoint. In contrast, the unvaccinated group remained relatively stable, never exceeding 40% dissimilarity in any region. Irregular patterns observed in the unvaccinated group’s first batch (ORF3, ORF5, and ORF7) were likely due to low sequencing quality, as also observed at the consensus level (Fig. 4a).

ORF5 exhibited distinct patterns, with Vac.Ing compositions remaining closest to the challenge virus throughout the experiment, while Unvac and Vac.Pre groups showed greater dissimilarity in later batches. However, overall dissimilarity in ORF5 remained below 35% (excluding the problematic unvaccinated Batch 1), highlighting reduced quasispecies shifts in this region (Fig. 4a).

These patterns are statistically supported by beta regression models of the dissimilarity index between individual samples and the challenge virus. Both nsp11 and ORF3 showed significant positive interactions between vaccinations and batch progression (Vac.Ing × Batch and Vac.Pre × Batch: *P* < .001 to *P* < .05), confirming accelerating divergence in vaccinated groups over time. ORF2a exhibited a significant positive effect for Vac.Pre group (*P* < .05), while ORF7 showed a significant positive effect for Vac.Ing (*P* < .01) and batch effect (*P* < .01), with a significant negative Vac.Ing × Batch interaction (*P* < .01) reflecting the plateau in dissimilarity after initial increases. ORF5 showed no significant vaccination, batch, or interaction effects, though it exhibited significant time-dependent divergence (dpi effect: *P* < .001). Despite technical factors (full read depth before down-sampling and Ct values) were significant for some regions (*P* < .05 to *P* < .001), their effect magnitudes were substantially smaller than treatment effects (Table 2).

**Table 2:** Coefficients of variables in the beta regression models for the Bray–Curtis dissimilarity index between individual samples and the challenge virus across observed genomic regions. Separate models were fit for each gene/region.

Gene/region	Vac.Ing	Vac.Pre	Batch	Vac.Ing × Batch	Vac.Pre × Batch	dpi	Ct	Full read depth	
nsp11	−0.6857	−0.5479	−0.0934	0.9464^***^	0.7354^***^	0.0359	−0.0424	−0.0001
ORF2a	0.5684	1.7984^*^	0.3288	0.2735	0.372	0.0693	0.0129	0.0005^*^
ORF3	0.5273	0.484	0.2649	0.5219^**^	0.4061^*^	0.0615	−0.0133	−0.001^***^
ORF5	0.2717	−1.2339	−0.1716	−0.2437	0.3907	0.2197^***^	0.0636	0.0005
ORF7	2.236^**^	0.7933	0.4248^**^	−0.5747^**^	−0.1307	0.0211	0.0179	−0.0006^*^

Significance of coefficients: ^*^P < .05; ^**^P < .01; ^***^P < .001.

Focusing on within-batch comparisons for each group, the Vac.Pre group exhibited the highest dissimilarities in OTU composition patterns, which were generally higher than those of the other groups during the middle batches but sharply decreased in the later batches. The Vac.Ing group consistently showed a downward trend in within-batch dissimilarity towards the endpoint, whereas the Unvac group displayed a slight upward trend as the experiment progressed (Fig. 4b).

PERMANOVA and multilevel pairwise comparisons were conducted to evaluate differences in OTU composition dissimilarity across groups and batches for each genomic region. Except for ORF5, the OTU compositions of the Unvac group were significantly different from those of both vaccinated groups (*P* = .001). Additionally, the composition of Batch 6 (the final batch) was significantly different from that of at least one of the early batches (Batches 1–3, *P* = .001–.002). Significant differences were also observed between the Vac.Ing and Vac.Pre groups in all regions (*P* = .001–.006), except for ORF7. For ORF5, significant differences were only detected between the Vac.Ing group and the other two groups (*P* = .001–.006), with no significant differences across batches. It is important to note that these comparisons rely on the assumption of homogeneous dispersion of dissimilarities within the compared variables (group or batch). However, this assumption was violated in most of our data, except for the comparisons between the Unvac group and both vaccinated groups in ORF7 (Supplementary Table 6). As a result, unequal dispersions may have confounded the differences identified by the test, and outputs of the statistical tests should be interpreted with caution.

### Site-wise selection pressures measured on viral quasispecies were not consistently higher in vaccinated groups

The proportion of amino acid sites under purifying or diversifying selection pressure did not differ between treatment groups for GP2 (encoded by ORF2a; 8.6%–9.4% of total sites) and GP3 (encoded by ORF3; 6.7%–8.7% of total sites). Interestingly, purifying selection in GP5 (encoded by ORF5) and N (encoded by ORF7) proteins was strongly evident in the Vac.Pre group (22%–23% of total sites), with more than twice the number of amino acid sites under selection compared to the Unvac (1.6%–8% of total sites) and Vac.Ing (3.5%–6.5% of total sites) groups for the same proteins. Contrary to expectations, these selective pressures were not predominantly localized within known immunologic epitope sites identified in previous studies (Wang et al. 2014, Popescu et al. 2017, Han et al. 2019). The median percentage of selected sites within a gene’s epitope relative to the total selected sites—calculated across the three genes with defined epitopes (GP2, GP3, and GP5)—was 12.5% (IQR: 9.38%–22.92%) for the Unvac group, 8.7% (IQR: 5.31%–13.44%) for the Vac.Pre group, and 18.18% (IQR: 9.09%–20.86%) for the Vac.Ing group. The highest percentage of selected sites within an epitope was 33.34% (7/21) in GP3 of the Unvac group, comprising 50% (6/12) purifying-selected sites and 11.11% (1/9) diversifying-selected sites. Conversely, the lowest percentage, 0%, was observed in GP5 of the Vac.Ing group (Supplementary Figs 5 and 6). Although many selected sites fell outside defined epitopes, some may still have functional importance; for instance, diversifying selection was detected at residue 151 of GP5 in the Vac.Pre group, a site known to be related to the virus virulence (Allende et al. 2000). A complete list of all selected sites is provided in Supplementary Table 7.

## Discussion

The infection chains of the IA/2014 PRRSV-2 isolate in vaccinated and non-immunized animals, as observed in this study, provide detailed insights into the virus’s evolutionary dynamics, spanning from consensus genome changes to within-host viral subpopulations (viral quasispecies). The analyses consistently revealed greater divergence of the virus in vaccinated animals compared to non-immunized animals under identical experimental conditions. At the genome level, the amount of genetic changes observed in viruses from the vaccinated groups was at least twice as high as the genetic change in the non-immunized group by the study’s endpoint. Furthermore, within-batch OTU dissimilarity indices showed that the diverged viruses within vaccinated groups were more uniform, whereas the viruses in the non-immunized group retained variability as the infection chain progressed. However, the amino acid mutation patterns were not consistent with selection pressure specific to known immunologic epitope sites.

The effect of modified live virus (MLV) vaccination on PRRSV evolution has been explored in several field studies, where external factors might have influenced the outcomes. Findings from these studies include: an increase in structural gene heterogeneity and diversifying selection of PRRSV-1 within 3 years following the introduction of the first PRRSV-1 vaccine in different regions of Korea (Kwon et al. 2019); an increase in genetic heterogeneity and the emergence of new phylogenetic clusters of PRRSV-2 ORF5 within a year of implementing the Ingelvac MLV programme in a mixed PRRSV-1- and PRRSV-2-infected farm in Thailand (Nilubol et al. 2014); and multiple turnovers of newly emerging PRRSV-1 variants, including mutations at key amino acid sites, within 1.5 years in a vaccinated herd in Spain (Clilverd et al. 2024). As field studies conducted at the population level, these studies lack control groups to assess causality. In contrast, our controlled experiment minimized potential confounding factors affecting immune response or virus evolution, such as variation in farm characteristics and biosecurity practices, host genetics (Rowland et al. 2022), complex vaccination programmes, co-infection with other pathogens (Chrun et al. 2023), diverse modes of transmission, the introduction of external variants, or recombination between wild type and vaccine strains (Li et al. 2009, Wang et al. 2019). In this experiment, the vaccine was the sole independent variable and the primary factor evaluated for its effect on the outcome. The evolutionary dynamics observed in vaccinated animals in our study closely align with the field studies, including increased heterogeneity relative to the original virus, the diversification of new phylogenetic clusters, and the succession of novel variants in later host populations.

The two MLV vaccines used in this study represent widely utilized commercial vaccines in the USA: Prevacent® PRRS MLV, belonging to sublineage 1D (variant 1D.2), and Ingelvac PRRS® MLV, belonging to sublineage 5A (variant 5A.1), based on ORF5 classification (Yim-im et al. 2023, VanderWaal et al. 2024). Meanwhile, the virus used as the challenge virus in this study is one of the current predominant US strains, sublineage 1A (variant 1A-unclassified) (Schroeder et al. 2021). In terms of overall evolutionary dynamics of the challenge virus, our results did not reveal a significant difference between the two vaccinated groups, despite the Prevacent vaccine presumed to be more homologous to the challenge virus (both belong to lineage 1). This finding may be explained by two reasons. First, there is no general consensus on what constitutes homologous *versus* heterologous PRRSV-2 strains based on ORF5 gene similarity, and the level of cross-protective immune response can vary considerably (Murtaugh and Genzow 2011, Charerntantanakul 2012, Li et al. 2014, Proctor et al. 2022). Second, the exact genetic distances between the vaccines and the challenge virus were only marginally different (ORF5: 12.2% for Prevacent, 14% for Ingelvac; WGS: 18.3% for Prevacent, 17.4% for Ingelvac), potentially leading to a similar level of cross-protection given that both vaccine viruses were quite distant from the challenge virus. Nevertheless, viruses evolving under partial immunity from the two vaccines diverged from each other and the original challenge virus. This divergence was evident from the consensus genome distance matrix (Fig. 2b) and the dissimilarity index of viral quasispecies composition in each gene (Supplementary Table 3), highlighting distinct viral subpopulations that may have escaped host immunity conferred by each vaccine.

Selection under immune pressure, where viruses capable of evading host immunity are more likely to persist and transmit to other hosts, provides a plausible explanation for the findings in this study. Our experimental results demonstrated this phenomenon by revealing that viruses collected from both vaccinated groups, although highly diverged from the original challenge virus to the extent that no founding OTU subpopulations remained, exhibited reduced within-group diversity at the endpoint. In contrast, the non-immunized group maintained a consistently diverse viral subpopulation throughout the course of the experiment. However, it is important to note that this selective pressure was likely amplified by the experimental design and may not occur as rapidly under natural field conditions. Specifically, animals in subsequent batches were infected not through natural transmission routes but *via* intramuscular inoculation of pooled serum with the lowest Ct values from animals of the previous batch, enabling infection even where shedding may have been low, particularly in vaccinated groups.

A quantitative analysis of amino acid mutations at the quasispecies level suggested a strong role of purifying selection pressures, which eliminate deleterious mutations that reduce viral fitness. This was particularly evident in the GP5 and N proteins of viruses from animals immunized with the Prevacent vaccine. This observation aligns with the hypothesis that purifying selection is the primary evolutionary force driving population homogeneity (Nowak and Schuster 1989, Moya et al. 2000), a feature observed in the later batches of vaccinated animals. However, site-wise selection pressures associated with immunologic epitopes were relatively infrequent compared to field studies, where diversifying selection is frequently reported in hypervariable regions (residues 32–36 and 57–61) flanking the broad neutralizing epitope of the GP5 protein (Hu et al. 2009, Delisle et al. 2012, Do et al. 2016, Paploski et al. 2019, Rupasinghe et al. 2022). This discrepancy likely stems from our study’s focus on within-host quasispecies, which considers even low-frequency subpopulations over a limited timeframe, whereas field studies typically examine successful viral variants responsible for outbreaks in large pig populations over several years.

Despite this difference, some selected sites in antigenic regions from our study are consistent with a prior pig-to-pig passage experiment using VR-2332 (the PRRSV-2 prototype) as the inoculum, which identified dN mutations at residues 33 and 34 of GP5 (Chang et al. 2002). These mutations potentially induced N-linked glycosylation, leading to glycan shielding of the neutralizing epitope (Ansari et al. 2006). In our study, diversifying selection was detected at residue 34 of the Prevacent group’s GP5. Additionally, analysis revealed that residue 151 of GP5 in the Prevacent group was under diversifying selection (detected by FUBAR and MEME). This residue is known to exhibit frequent amino acid reversions during cell culture passage and may influence structural changes in the GP5-M heterodimer, which may be associated with PRRSV-2 attenuation or virulence (Storgaard et al. 1999, Allende et al. 2000, Chang et al. 2002). Furthermore, diversifying selection was detected at residue 58 of GP5 in the non-immunized group, a site located within hypervariable region 2 and epitope C, which is a target of homologous neutralization (Popescu et al. 2017) and is frequently mutated within herds experiencing repeated outbreaks caused by a single virus (Baker et al. 2025). The critical sites addressed here are based on previous findings that exclusively examined GP5 and represent only a small subset of widely distributed residues under selection pressure found in this study. To confirm that selection at these sites is genuinely associated with immune pressure from the vaccine and the impact of these changes on immune recognition, further studies of longer duration and with larger sample sizes are necessary.

Comparing the evolutionary dynamics of PRRSV-2 at both the genome-wide and gene-specific levels suggests that not all genes evolved in a coordinated manner or contributed equally to genome-level evolution. Consensus genome analysis revealed that several nsp coding regions in ORF1a and ORF1b exhibited high variability in both vaccinated groups, indicating that these genes contributed substantially to the observed genetic divergence. Mutations or even deletions in these non-structural regions may have minimal effects on key viral functions, allowing these genes to tolerate higher mutation rates as long as they remain non-deleterious, similar to deletions frequently observed in nsp2 of PRRSV (Fang et al. 2004, Brockmeier et al. 2012, van Geelen et al. 2018) and in many accessory genes of SARS-CoV-2 (Jeronimo et al. 2023). However, limited long-read sequencing depth in these regions restricted our ability to explore viral quasispecies diversity, and while normalized Shannon entropy accounts for coverage differences in quality-filtered genomes (see Methods), the observed variability patterns should be interpreted with caution as low depth may underestimate true diversity. In contrast, for structural protein-coding genes (ORF2 to ORF7), where quasispecies diversity could be assessed, some inconsistencies were observed among genes. These included variable batch effects on within-sample diversity and a distinct pattern of dissimilarity in quasispecies composition relative to the original challenge virus in ORF5 compared to other genes (i.e. unlike other genes, ORF5 quasispecies composition did not become increasingly dissimilar from the challenge virus across batches; Fig. 4a). Such discrepancies highlight the potential of different types of selection pressures operating on each gene, likely influenced by their functional roles.

The analytic approaches and result interpretation of this study were mainly limited by the unexpectedly low viral load in vaccinated groups. The initial sample size (*n* = 6–7) per batch per group was calculated under the assumption that the viral quantity from all animals would be sufficient to: (1) serve as an inoculum for the subsequent batch after pooling serum at the endpoint, (2) enable successful whole-genome sequencing, and (3) achieve adequate sequencing depth (>100 reads) for each genomic region of interest (Supplementary Table 2). However, during the experiment, we found that Ct values for some vaccinated animals at certain dpi exceeded 35, indicating clearance of PRRSV-2 infection. This pattern of reduced viral loads in vaccinated groups was observed consistently across batches, especially low in Batches 1 and 3 (Supplementary Figs 2 and 3), potentially affecting inoculum potency in subsequent transmission cycles. Consequently, adjustments were made to maintain the infection chain and modify sample selection for sequencing, as described in the Methods section. These changes led to unbalanced data, such as unequal sample sizes per batch per group and the exclusion of many genes from the quasispecies analysis due to insufficient read depth in some groups. Additionally, within-batch diversity was partly determined from samples collected at different dpi from the same animal, meaning that the observed diversity reflected both viral evolution across different infected animals and the temporal dynamics of the same viral population within an individual host. Despite these limitations, our descriptive analyses and statistical tests—although based on imperfect data—were still able to capture distinctive evolutionary dynamics between vaccinated and non-immunized animals, regardless of the vaccine strain. Moreover, while technical factors related to unequal viral loads (including Ct values, collection dates, genome coverage, and read depth) could affect diversity comparisons, statistical tests showed these effects were generally small and unlikely to influence diversity metrics beyond the main factors of vaccination and batch at both consensus genome and quasispecies levels.

As mentioned previously, vaccinated groups had significantly lower viral loads compared to the non-immunized group. Therefore, the rapid viral evolution observed in vaccinated animals in this study should not be interpreted as an increase in transmissibility, infectivity, or virulence, as the likelihood of high transmissibility or infectivity is presumably lower due to reduced viral quantity (Charpin et al. 2012, Pileri and Mateu 2016). However, viruses evolving through passages in vaccinated animals may eventually diverge into novel groups potentially spawning new genetic variants. If such a variant possesses high fitness and can outcompete other circulating strains, then it could instigate epidemic spreading events in pig populations.

## Conclusion

The effect of immune pressure from vaccination on PRRSV-2 evolution has been debated for over a decade, especially now, as novel viral variants continue to emerge despite intensive use of vaccination. Our study addressed this question by demonstrating the distinct evolutionary dynamics of PRRSV-2 IA/2014 (a current circulating variant within sublineage L1A PRRSV-2) in vaccinated versus non-immunized animals under controlled experimental conditions. Viruses inoculated into vaccinated animals evolved to a greater extent, as evidenced by increased overall genomic distance and fine-scale shifts in quasispecies composition compared to the original challenge virus. While selection pressure on key antigenic sites was not particularly evident and varied among different genes, and viral loads were relatively low in vaccinated animals, the likelihood of novel genetically distinct viruses emerging from vaccinated populations was higher than in non-immunized populations; such novel viruses would not necessarily have higher fitness, and fitness assessments were not performed here. Given these findings, scientists and animal health practitioners involved in PRRS control should consider this phenomenon when developing future vaccines and designing vaccination programmes.

## Supplementary Material

Supplementary_materials_clean_veaf056

Supplementary_tables_veaf056

## Data Availability

Raw sequencing reads for all PRRSV-2 samples sequenced in this study, including the challenge virus (IA/2014 PRRSV-2), are available in the NCBI Sequence Read Archive (SRA) under BioProject accession number PRJNA1224032. Corresponding sample accession numbers are provided in Supplementary Table 2.
