# Correction to: Experimental evidence of vaccine-driven evolution of porcine reproductive and respiratory syndrome virus type 2

**DOI:** 10.1093/ve/veaf092

**Published:** 2025-11-22

This is a correction to: Nakarin Pamornchainavakul, Igor A D Paploski, Dennis N Makau, Julia P Baker, Jing Huang, Clarissa P Ferreira, Cesar A Corzo, Albert Rovira, Maxim C-J Cheeran, Samantha Lycett, Andrea Doeschl-Wilson, Declan C Schroeder, Kimberly VanderWaal, Experimental evidence of vaccine-driven evolution of porcine reproductive and respiratory syndrome virus type 2, *Virus Evolution*, Volume 11, Issue 1, 2025, veaf056, https://doi.org/10.1093/ve/veaf056.

The title of this article has been corrected from ‘Experimental evidence of vaccine-driven evolution of respiratory syndrome virus type 2’ to ‘Experimental evidence of vaccine-driven evolution of porcine reproductive and respiratory syndrome virus type 2’. The publisher apologizes for the error.

