# Supplementary material for: Experimental evidence of vaccine-driven evolution of porcine reproductive and respiratory syndrome virus type 2
Source: Virus Evol. 2025 Jul 22;11(1):veaf056. doi: 10.1093/ve/veaf056 (PMC12360701; doi:10.1093/ve/veaf056)
Supplement: Supplementary_materials_clean_veaf056 [file supplementary_materials_clean_veaf056.docx]

**Supplementary materials**


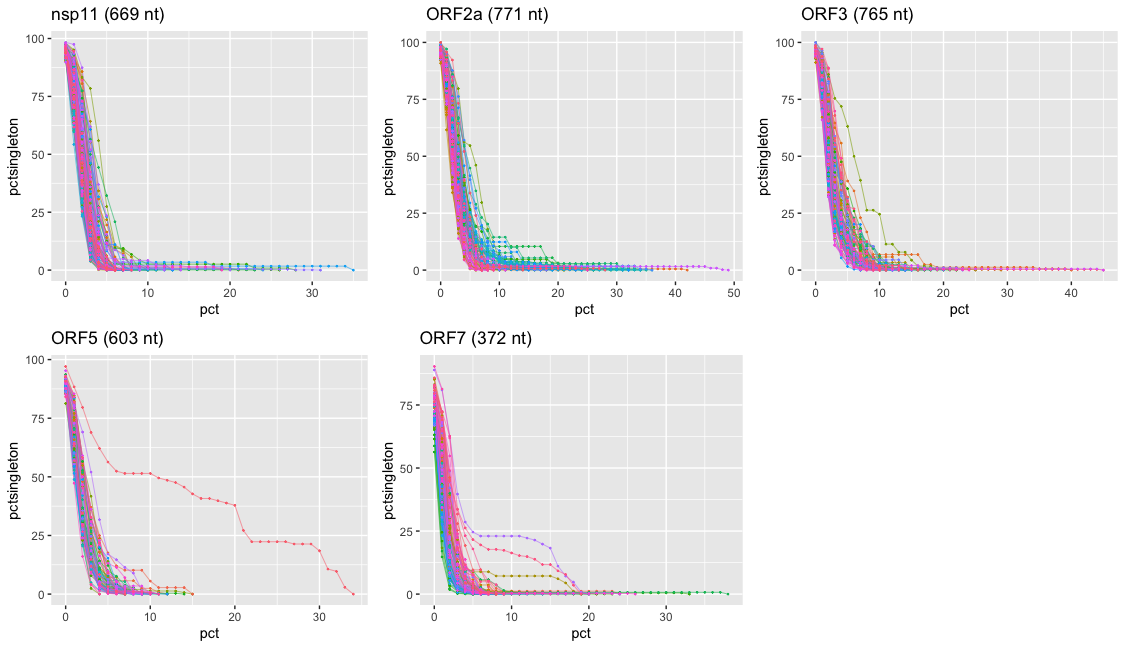


**Supplementary figure 1.** Percentage of singleton haplotypes (y-axis [pctsingleton]) in the sample based on different thresholds for sequencing noise minimization (x-axis [pct]: 0% indicates no replacement, while 1% indicates replacement of SNVs with a frequency lower than 1% of the total depth by the majority nucleotide at that position). A 5% threshold was chosen as it effectively reduced singleton haplotypes to an optimal level in most samples across all observed genomic regions.


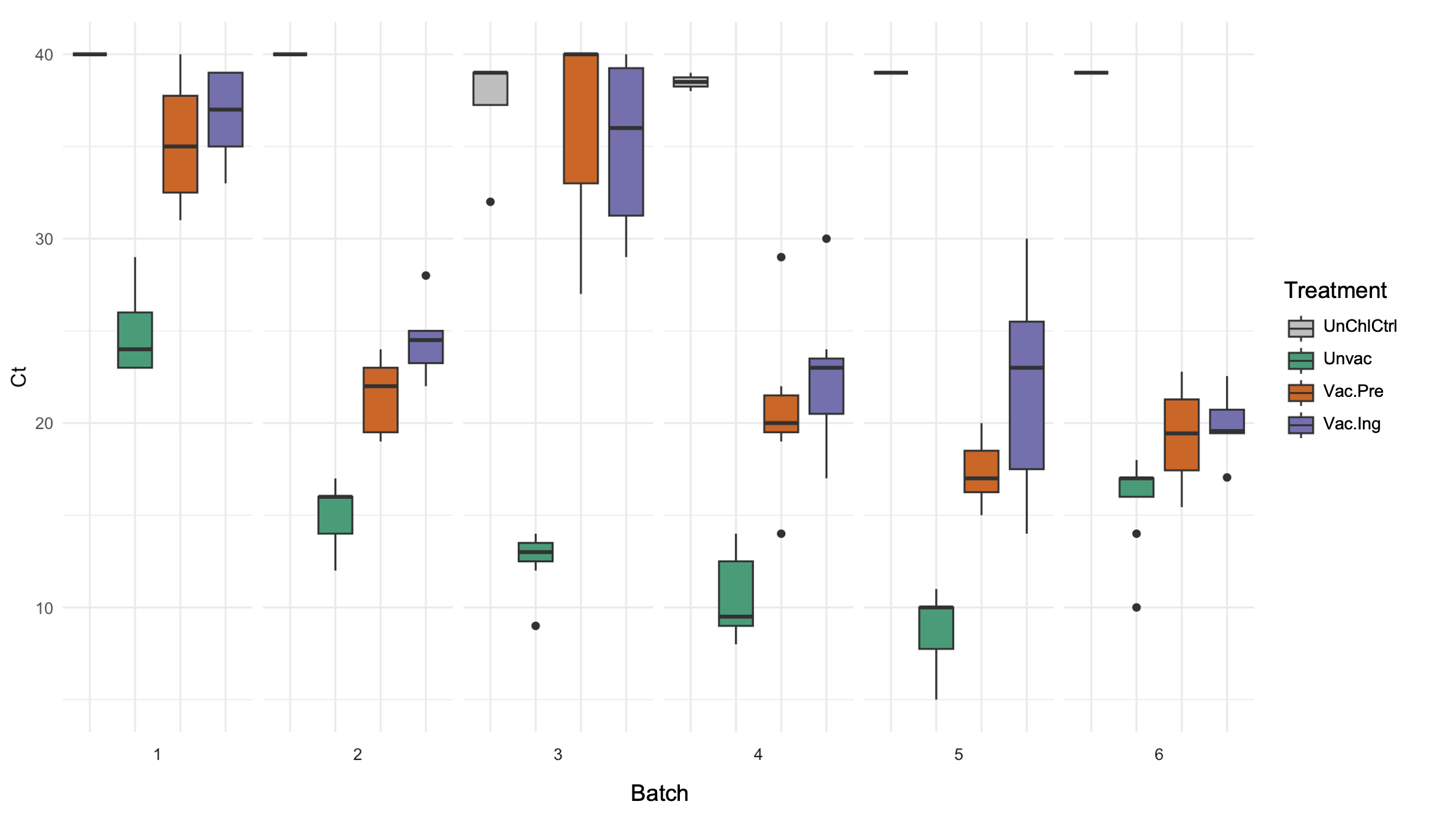


**Supplementary figure 2.** The lowest Ct values (from RT-PCR run at the UMN VDL) of PRRSV-2 from each animal by batch and group.


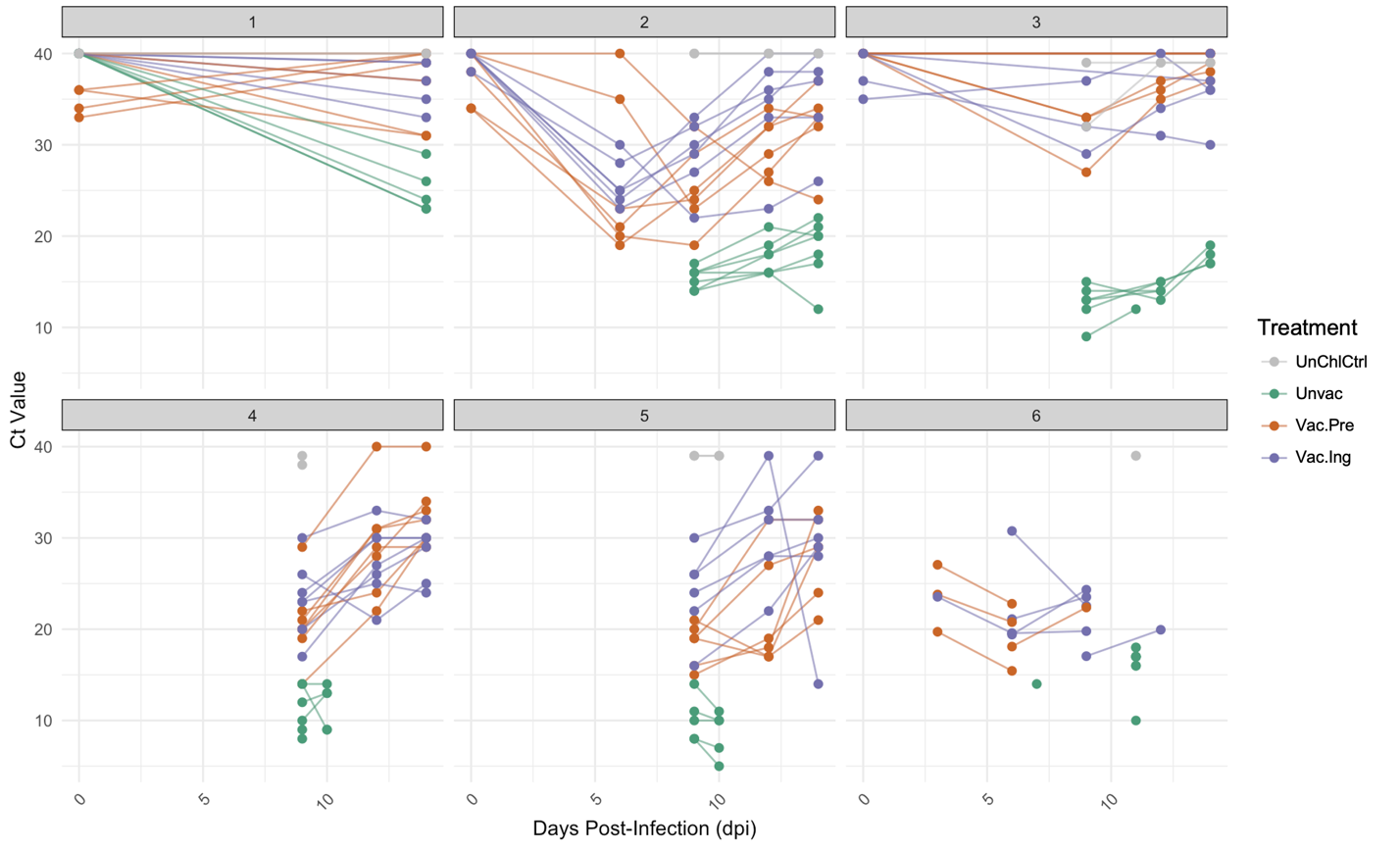


**Supplementary figure 3.** All available Ct values (from RT-PCR run at the UMN VDL) of PRRSV-2 from each animal by batch and group.


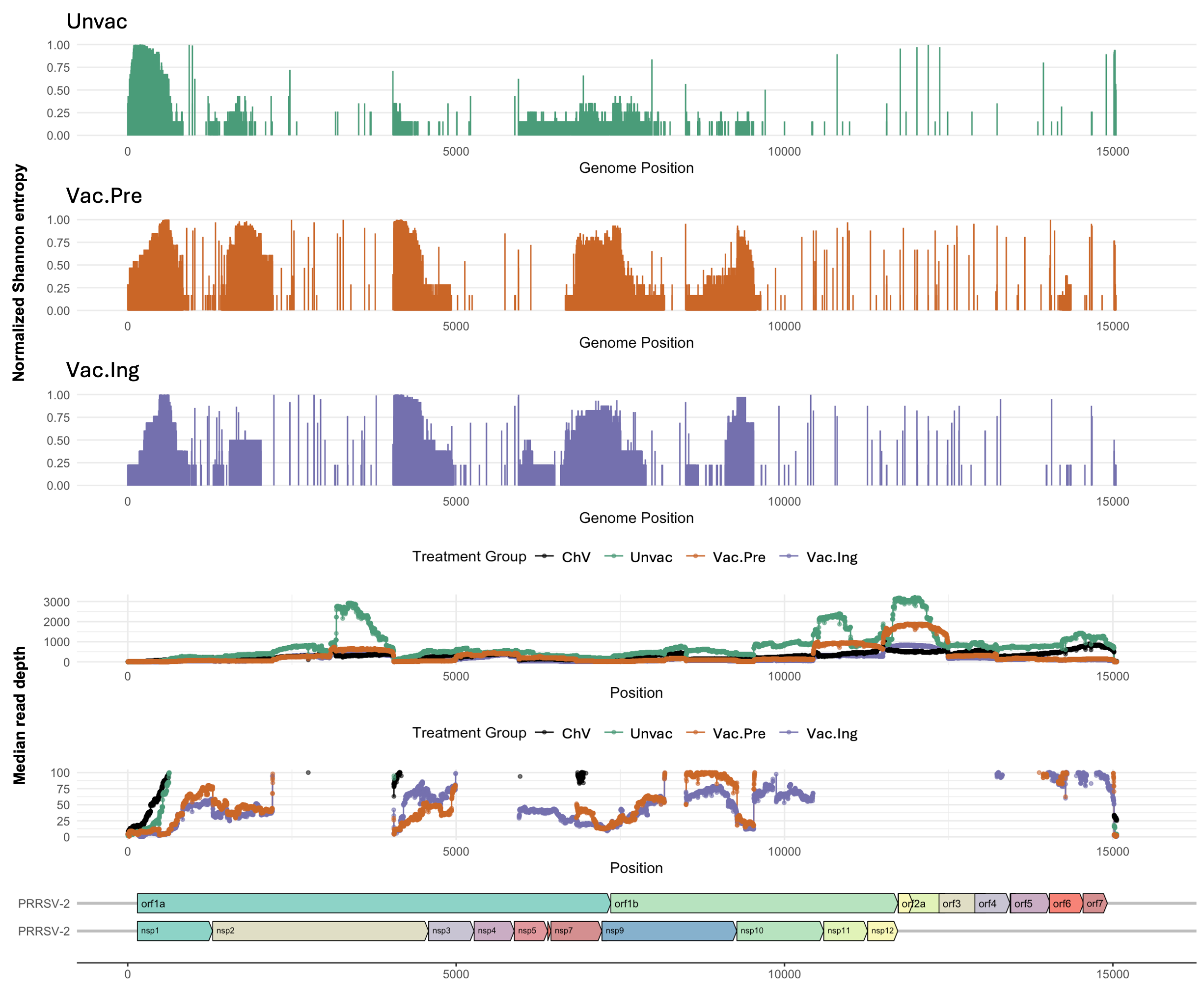


**Supplementary Figure 4.** Site-wise normalized Shannon entropy across PRRSV-2 genomes for each group (top). Mean sequencing depth per nucleotide position, with a zoom-in plot for depths below 100 (middle). PRRSV-2 genome annotation aligned with the above plots (bottom).


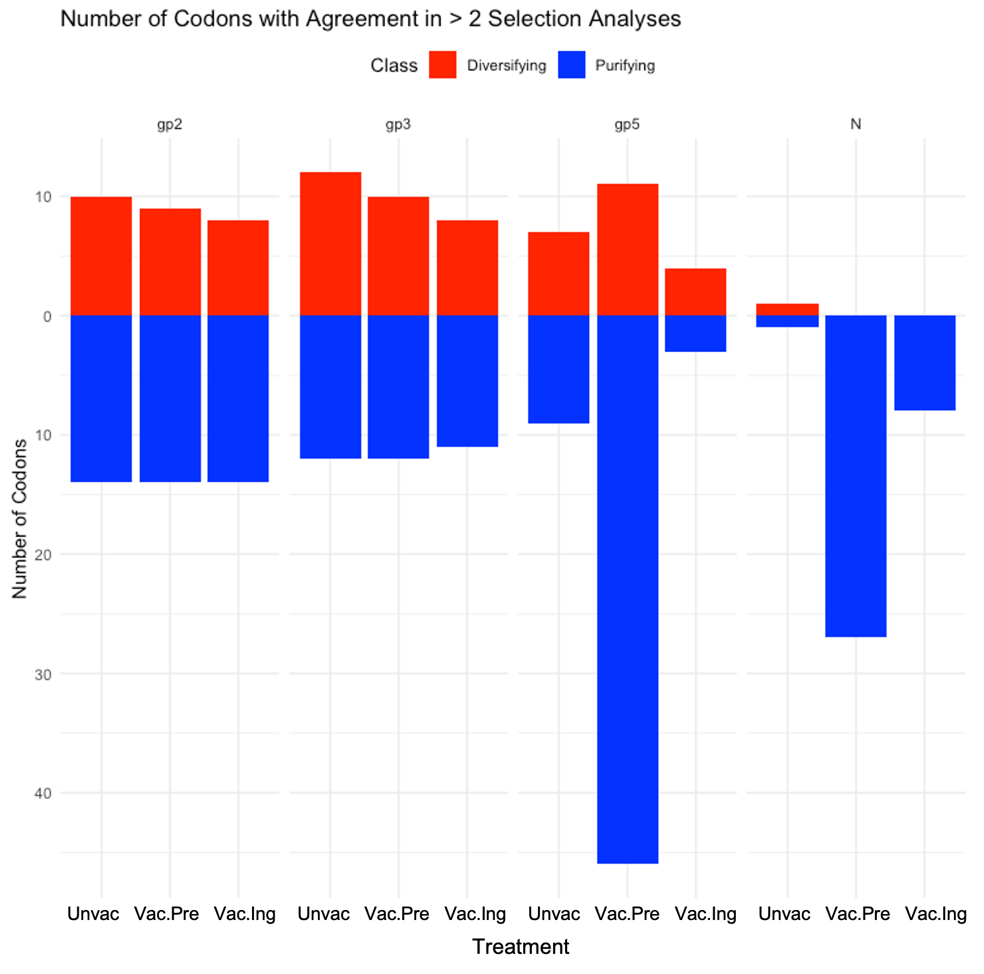


**Supplementary Figure 5.** Number of codons under selection pressure detected by at least two of the three methods (FEL, MEME, FUBAR), colored by type of selection (red = diversifying, blue = purifying).


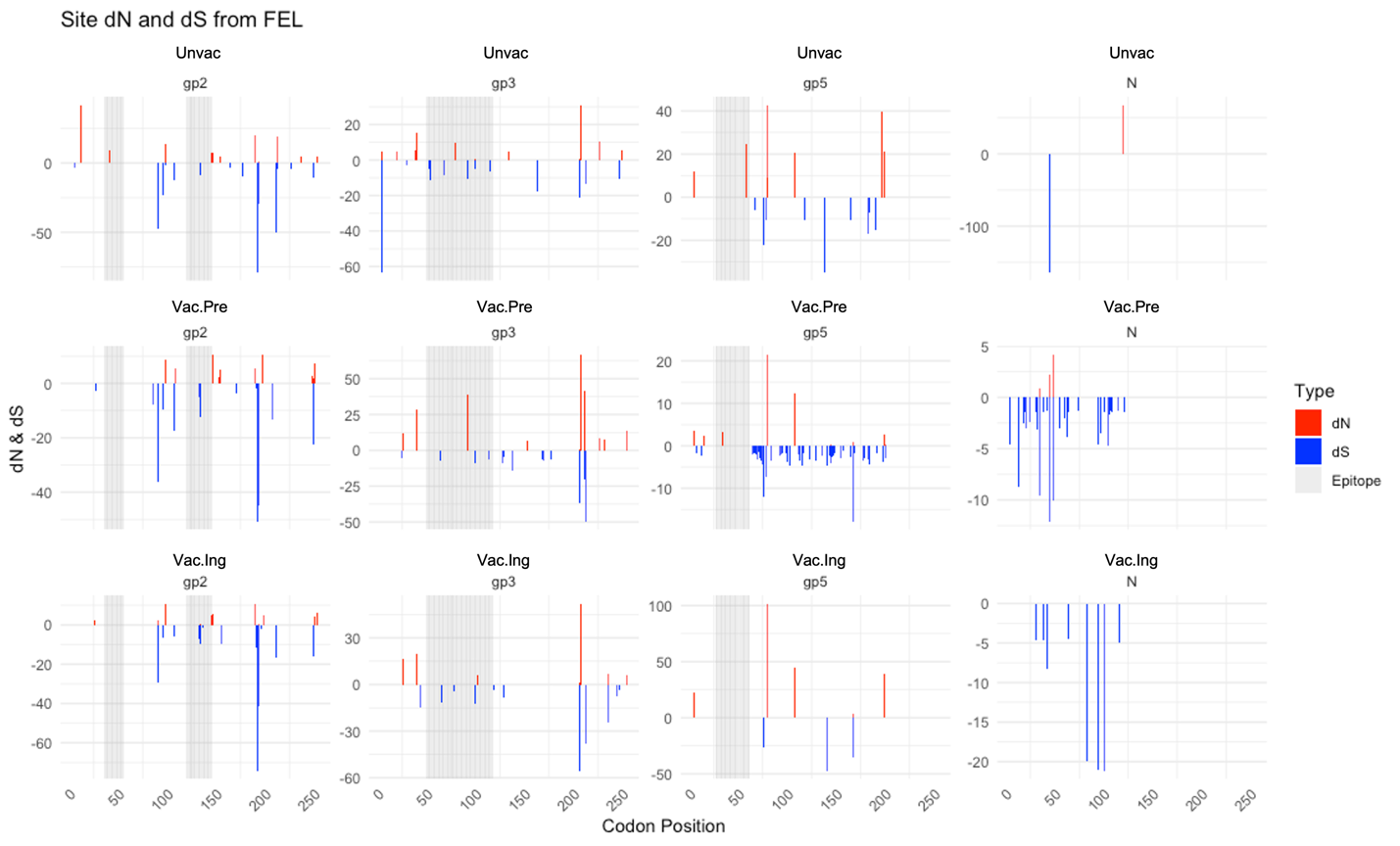


**Supplementary Figure 6.** Non-synonymous (dN) (red) and synonymous (dS) (blue) substitution rates from FEL analysis, presented by gene and group, with documented immunologic epitopes highlighted in grey.


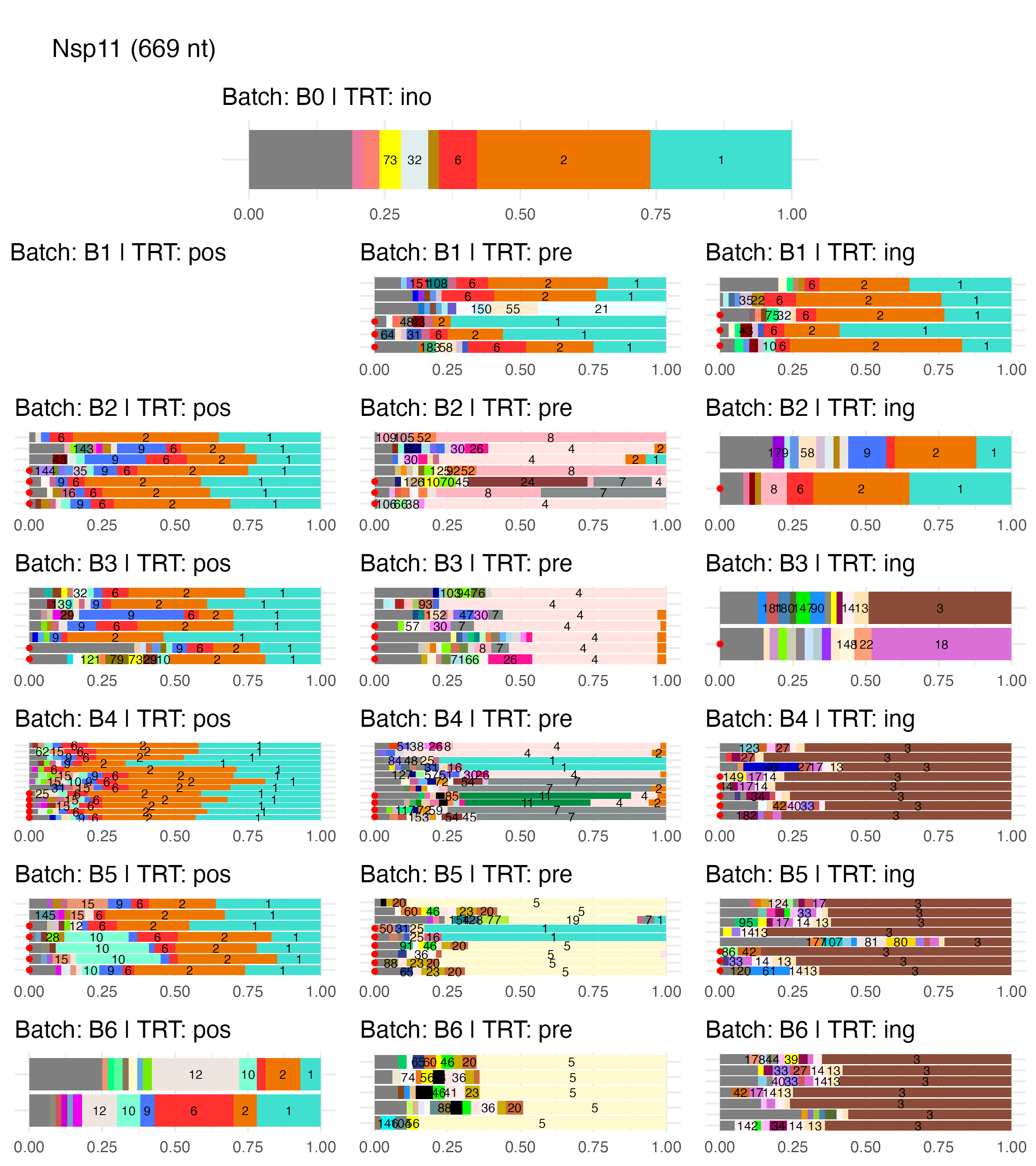


**Supplementary Figure 7.** Haplotypes of nsp11 region, represented by distinct colors and numbers, identified across treatment groups (ino: ChV; pos: Unvac; pre: Vac.Pre; ing: Vac.Ing) and batches. Red dots on the left side of each bar indicate samples derived from animals whose serum was used to produce the inoculum for the subsequent batch.


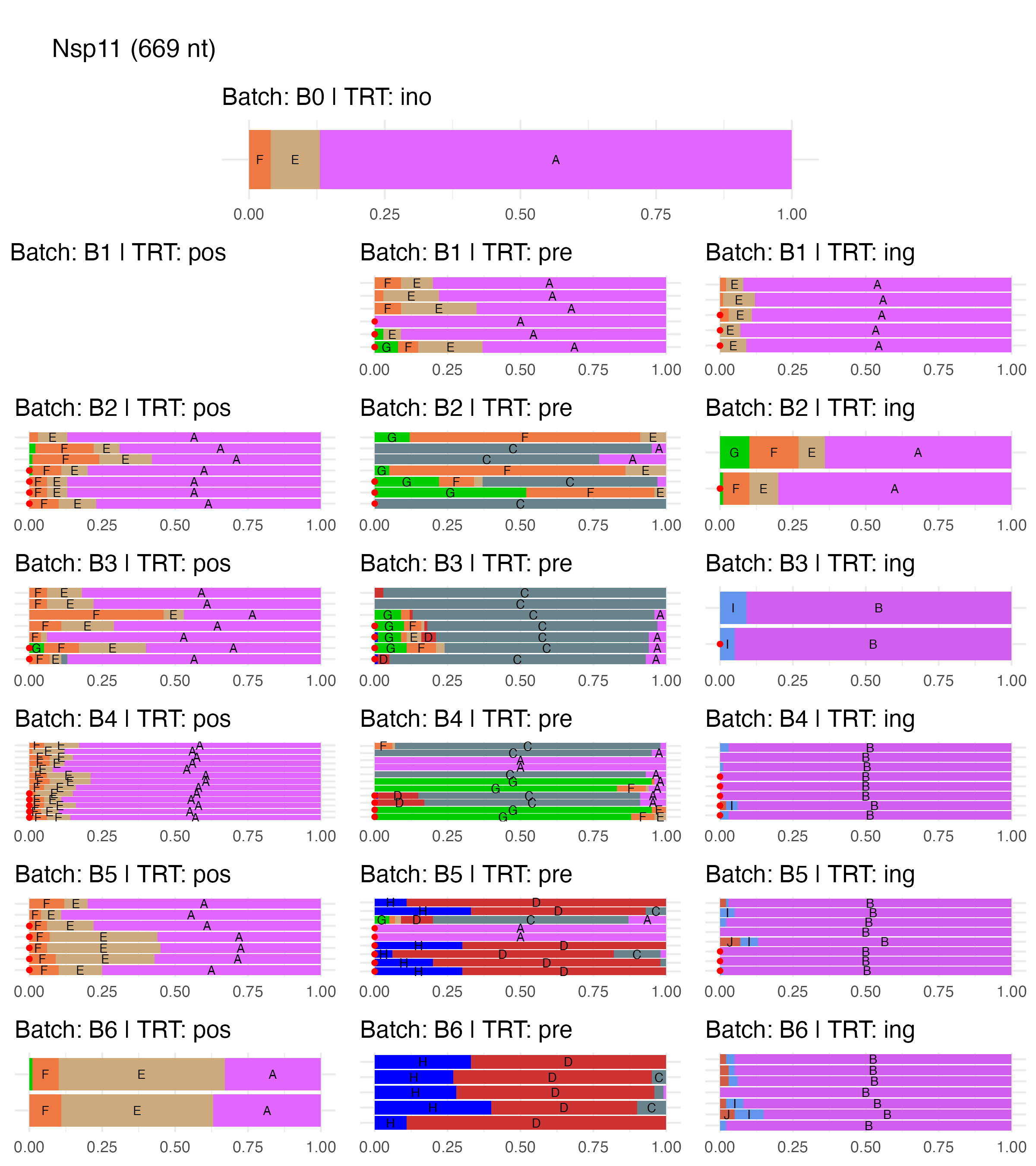


**Supplementary Figure 8.** OTUs of nsp11 region, represented by distinct colors and alphabets, identified across treatment groups (ino: ChV; pos: Unvac; pre: Vac.Pre; ing: Vac.Ing) and batches. Red dots on the left side of each bar indicate samples derived from animals whose serum was used to produce the inoculum for the subsequent batch.


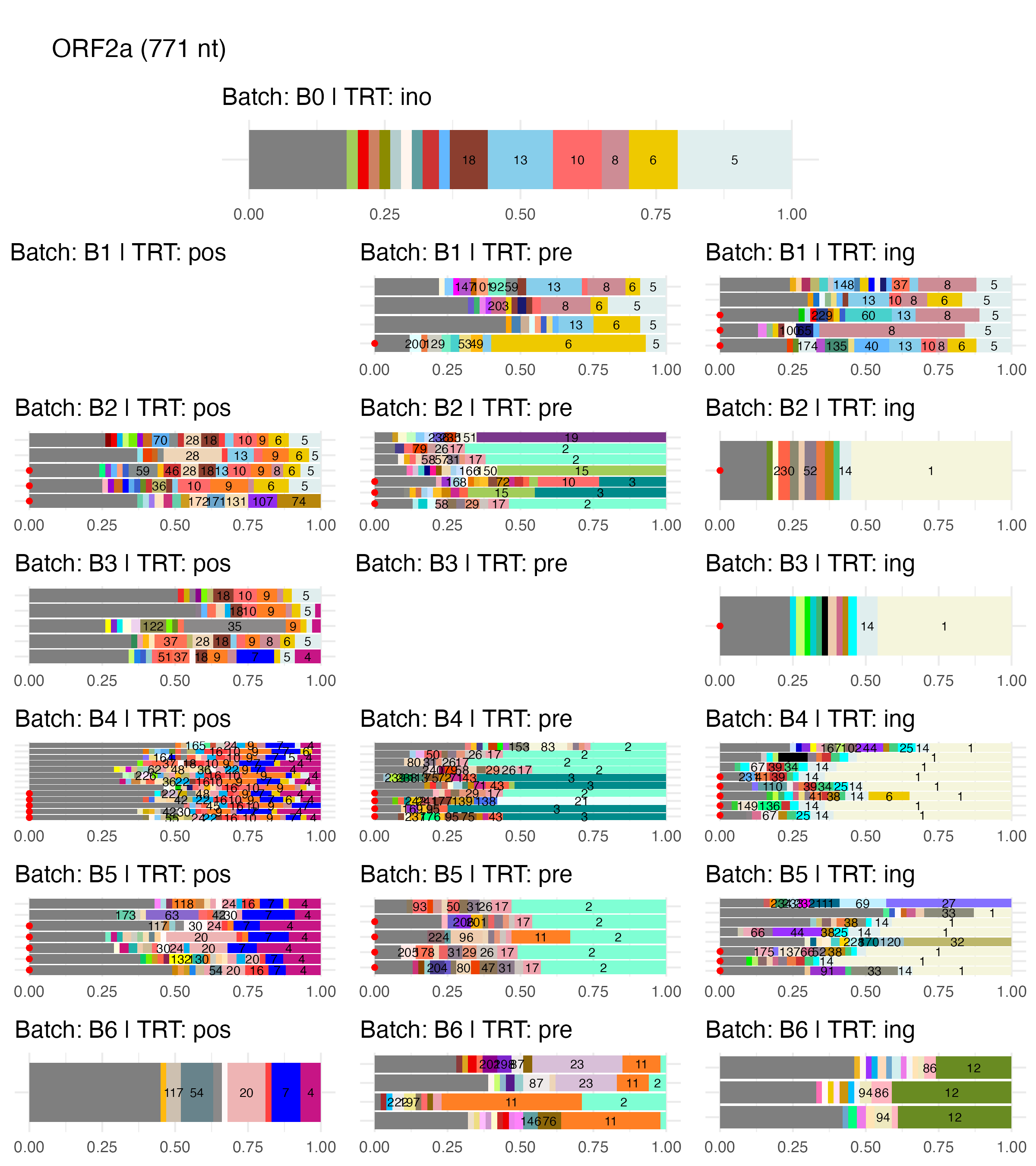


**Supplementary Figure 9.** Haplotypes of ORF2a gene, represented by distinct colors and numbers, identified across treatment groups (ino: ChV; pos: Unvac; pre: Vac.Pre; ing: Vac.Ing) and batches. Red dots on the left side of each bar indicate samples derived from animals whose serum was used to produce the inoculum for the subsequent batch.


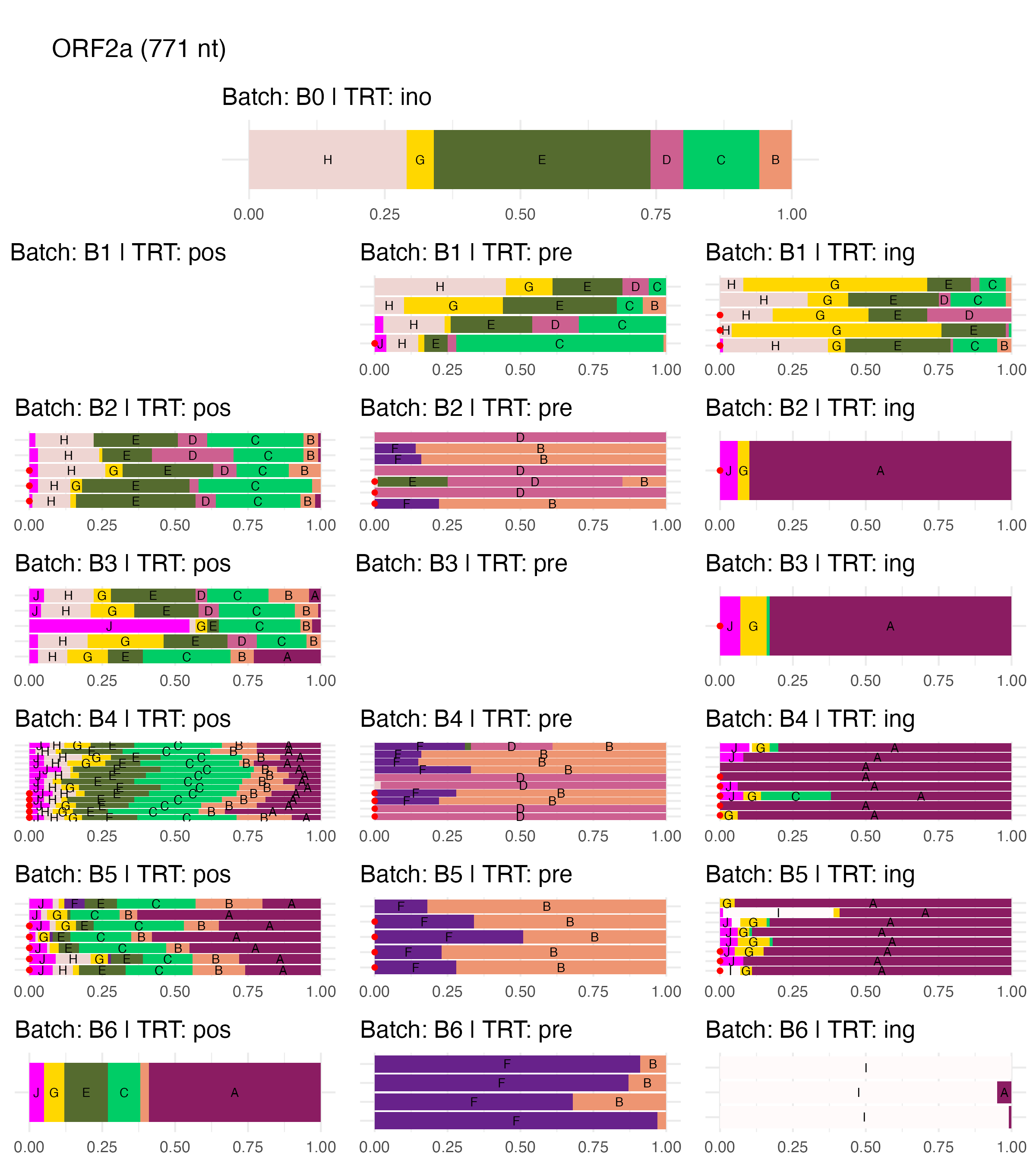


**Supplementary Figure 10.** OTUs of ORF2a gene, represented by distinct colors and alphabets, identified across treatment groups (ino: ChV; pos: Unvac; pre: Vac.Pre; ing: Vac.Ing) and batches. Red dots on the left side of each bar indicate samples derived from animals whose serum was used to produce the inoculum for the subsequent batch.


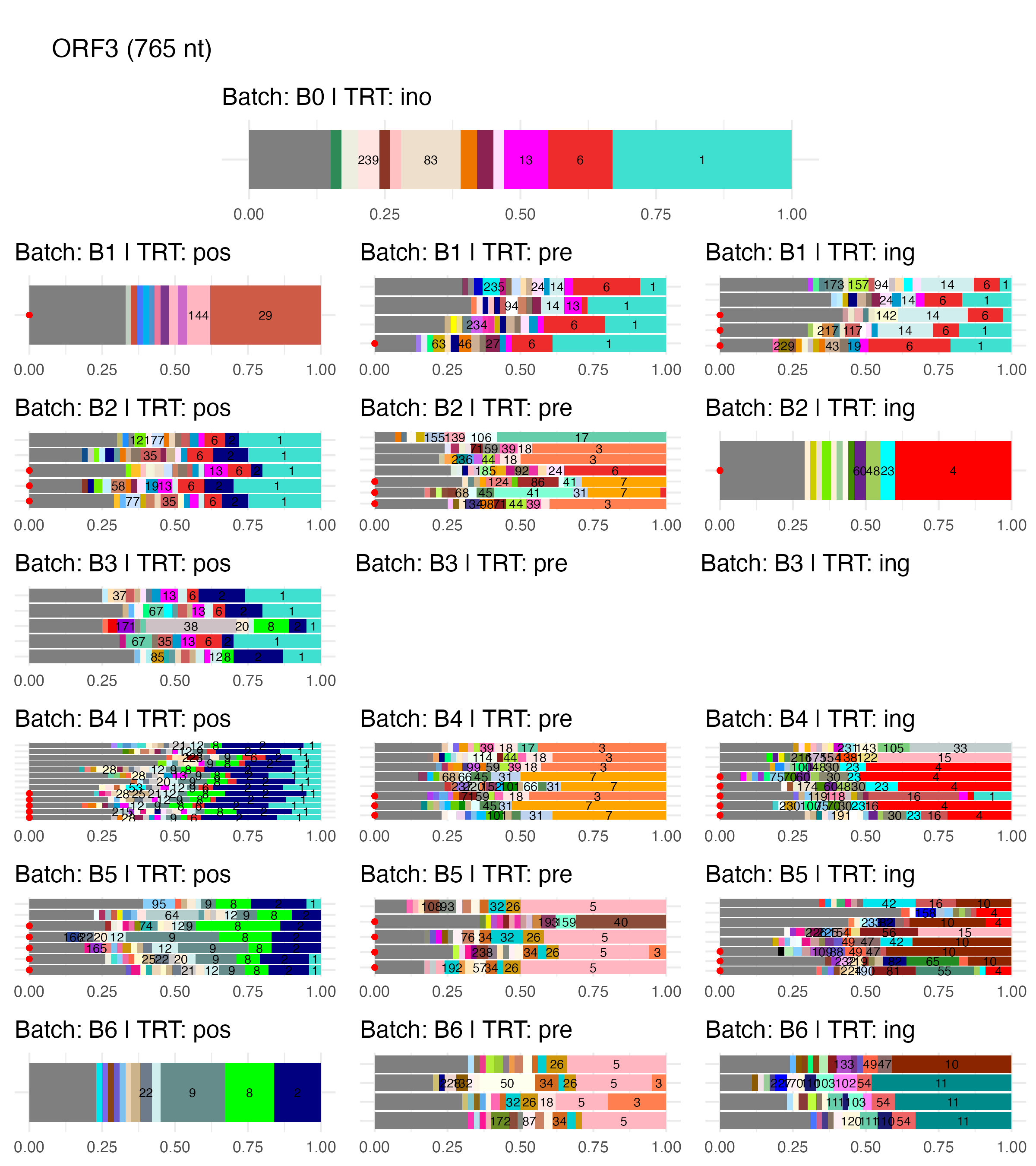


**Supplementary Figure 11.** Haplotypes of ORF3 gene, represented by distinct colors and numbers, identified across treatment groups (ino: ChV; pos: Unvac; pre: Vac.Pre; ing: Vac.Ing) and batches. Red dots on the left side of each bar indicate samples derived from animals whose serum was used to produce the inoculum for the subsequent batch.


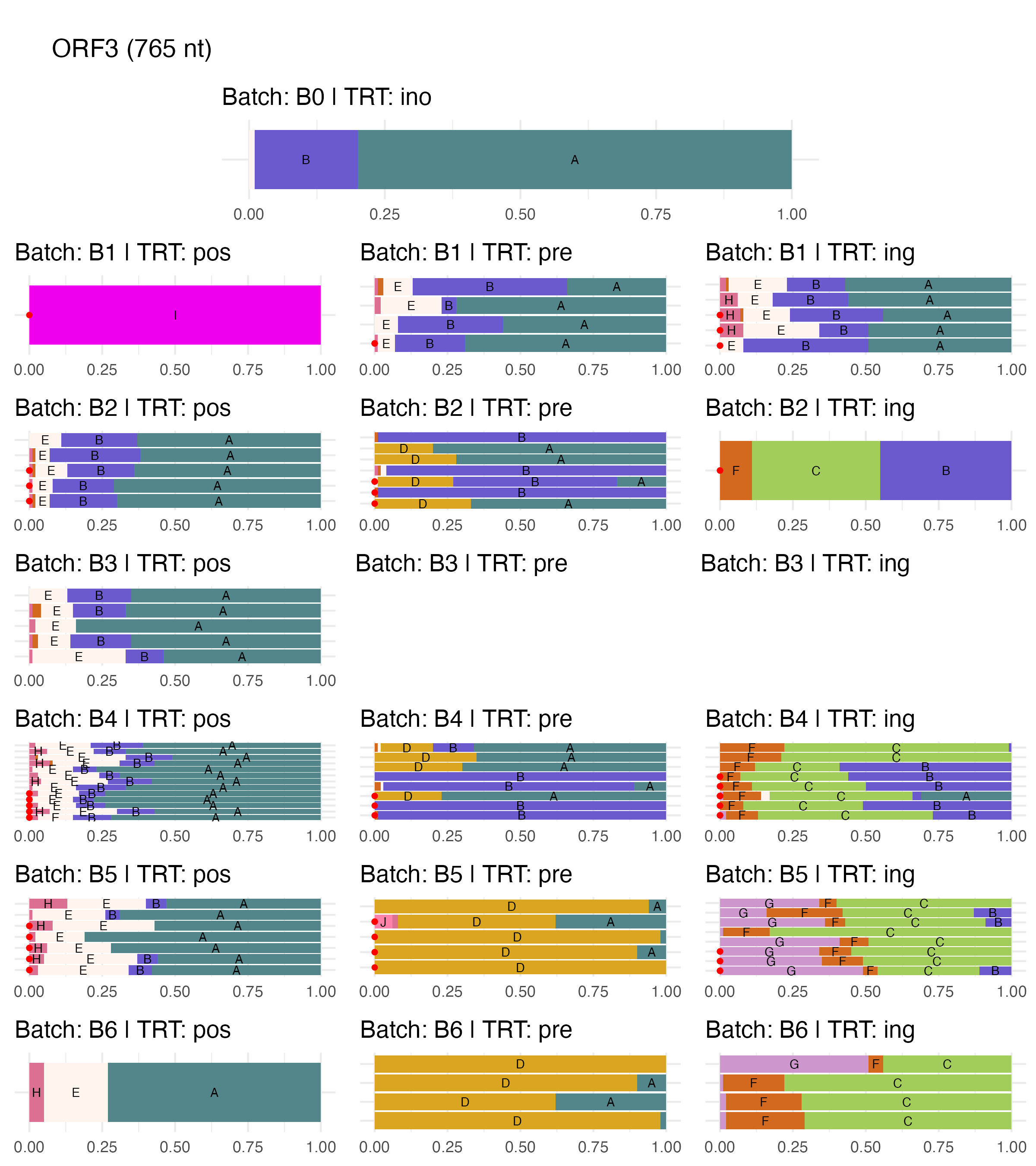


**Supplementary Figure 12.** OTUs of ORF3 gene, represented by distinct colors and alphabets, identified across treatment groups (ino: ChV; pos: Unvac; pre: Vac.Pre; ing: Vac.Ing) and batches. Red dots on the left side of each bar indicate samples derived from animals whose serum was used to produce the inoculum for the subsequent batch.


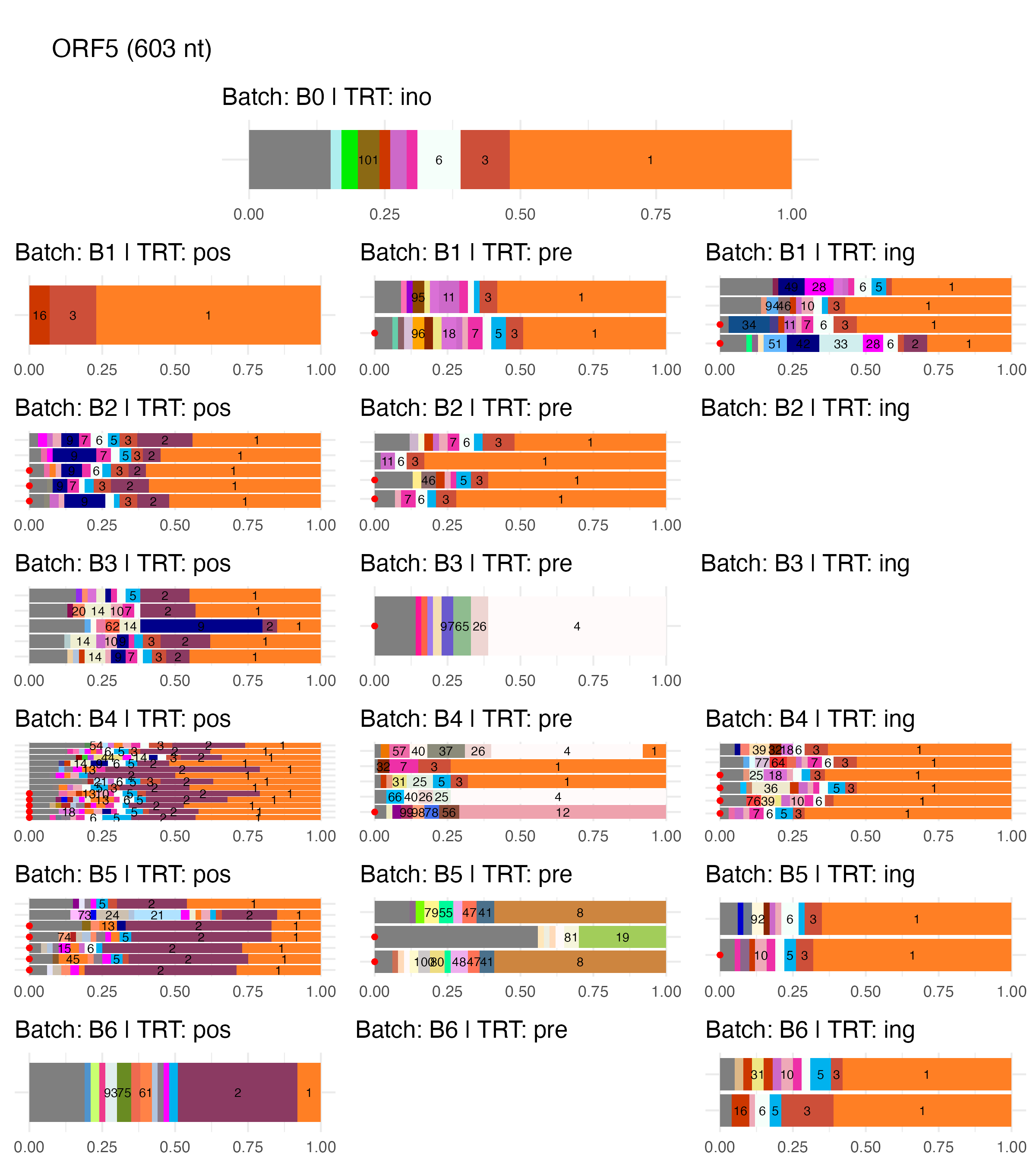


**Supplementary Figure 13.** Haplotypes of ORF5 gene, represented by distinct colors and numbers, identified across treatment groups (ino: ChV; pos: Unvac; pre: Vac.Pre; ing: Vac.Ing) and batches. Red dots on the left side of each bar indicate samples derived from animals whose serum was used to produce the inoculum for the subsequent batch.


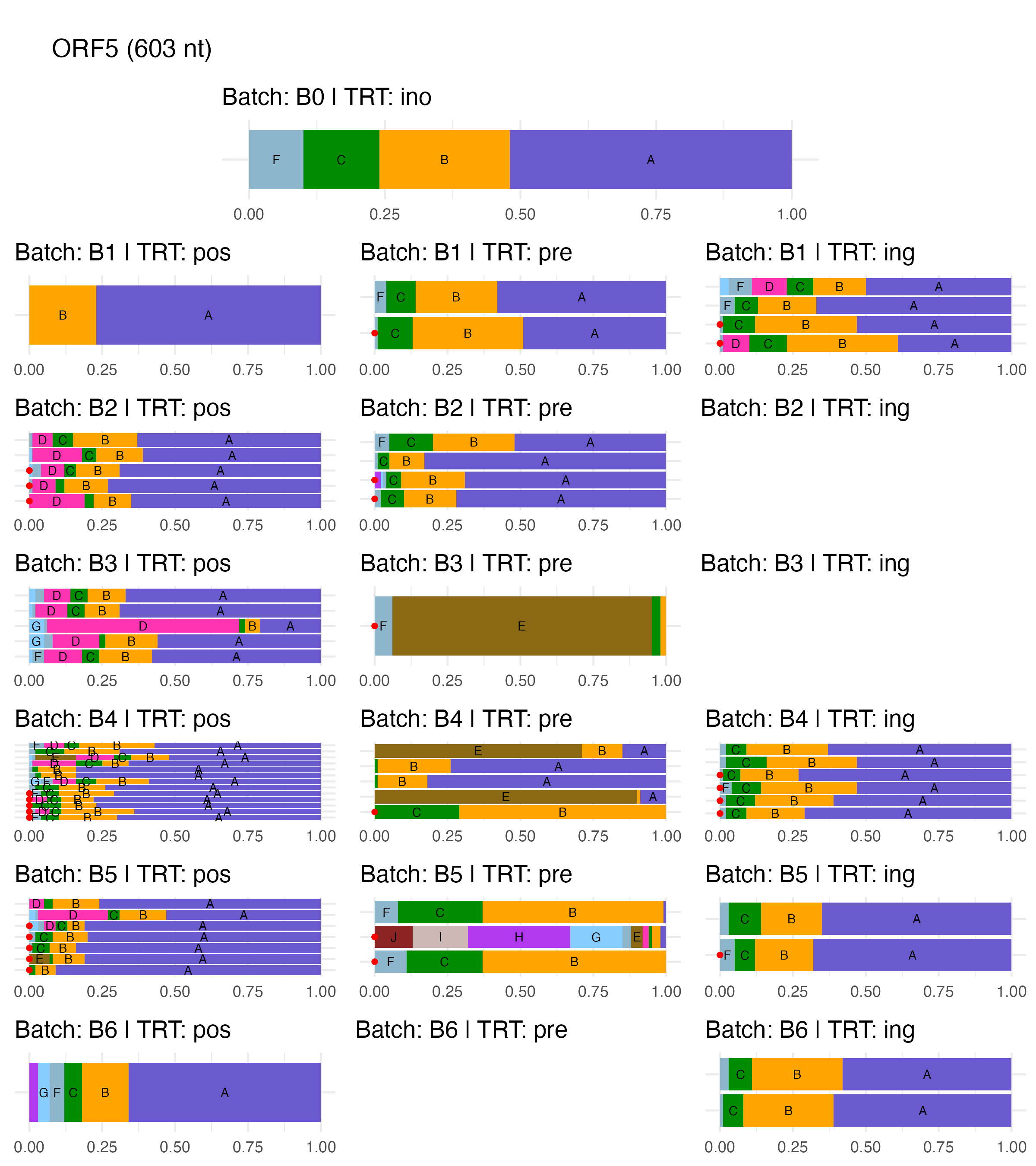
 **Supplementary Figure 14.** OTUs of ORF5 gene, represented by distinct colors and alphabets, identified across treatment groups (ino: ChV; pos: Unvac; pre: Vac.Pre; ing: Vac.Ing) and batches. Red dots on the left side of each bar indicate samples derived from animals whose serum was used to produce the inoculum for the subsequent batch.


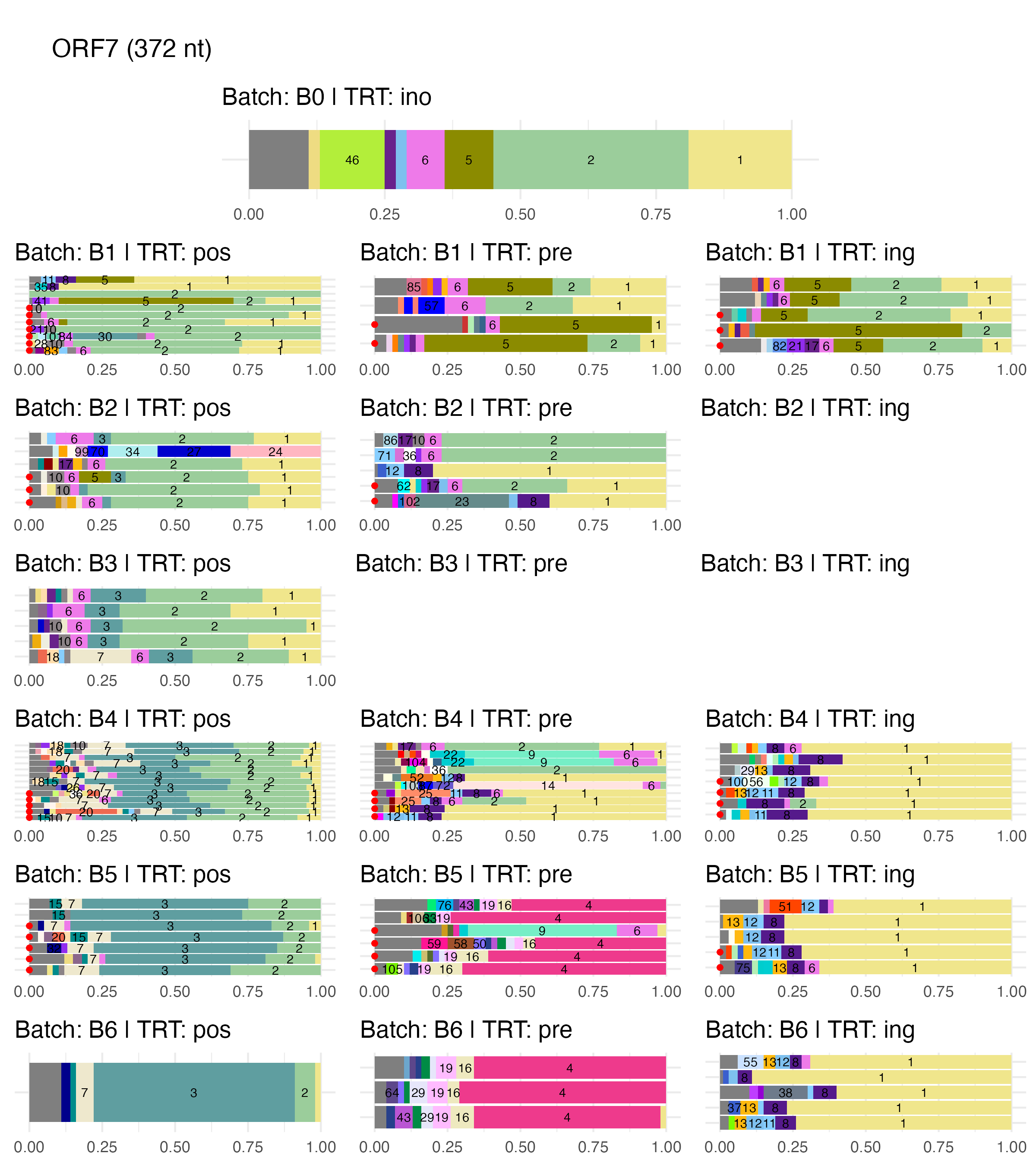


**Supplementary Figure 15.** Haplotypes of ORF7 gene, represented by distinct colors and numbers, identified across treatment groups (ino: ChV; pos: Unvac; pre: Vac.Pre; ing: Vac.Ing) and batches. Red dots on the left side of each bar indicate samples derived from animals whose serum was used to produce the inoculum for the subsequent batch.


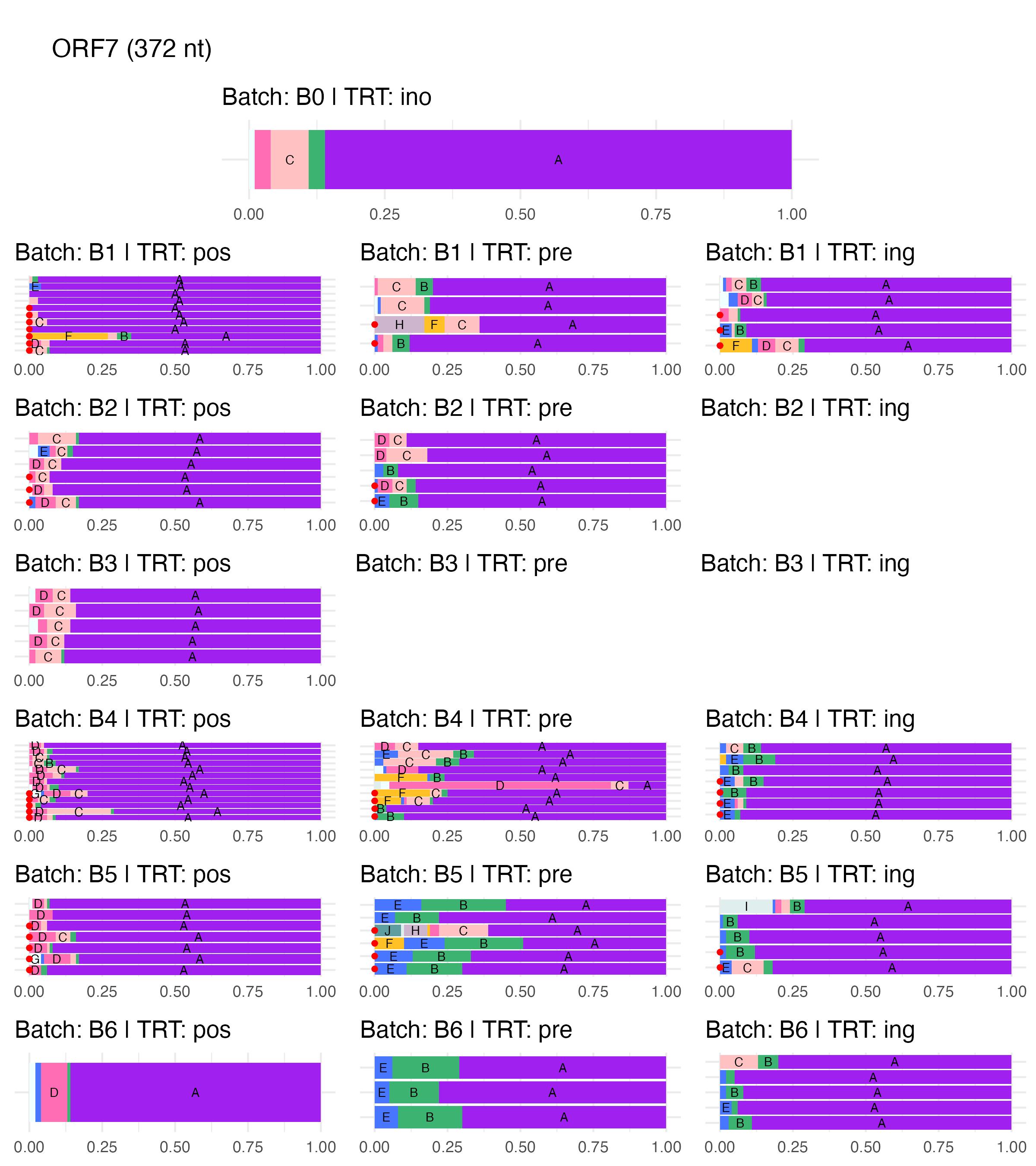


**Supplementary Figure 16.** OTUs of ORF7 gene, represented by distinct colors and alphabets, identified across treatment groups (ino: ChV; pos: Unvac; pre: Vac.Pre; ing: Vac.Ing) and batches. Red dots on the left side of each bar indicate samples derived from animals whose serum was used to produce the inoculum for the subsequent batch.
